# Accounting for reporting delays in real-time phylodynamic analyses with preferential sampling

**DOI:** 10.1371/journal.pcbi.1012970

**Published:** 2025-05-06

**Authors:** Catalina M. Medina, Julia A. Palacios, Volodymyr M. Minin

**Affiliations:** 1 Department of Statistics, University of California, Irvine, Irvine, California, United States of America; 2 Departments of Statistics and Biomedical Data Science, Stanford University, Stanford, California, United States of America; Ecole Normale Superieure, FRANCE

## Abstract

The COVID-19 pandemic demonstrated that fast and accurate analysis of continually collected infectious disease surveillance data is crucial for situational awareness and policy making. Coalescent-based phylodynamic analysis can use genetic sequences of a pathogen to estimate changes in its effective population size, a measure of genetic diversity. These changes in effective population size can be connected to the changes in the number of infections in the population of interest under certain conditions. Phylodynamics is an important set of tools because its methods are often resilient to the ascertainment biases present in traditional surveillance data (e.g., preferentially testing symptomatic individuals). Unfortunately, it takes weeks or months to sequence and deposit the sampled pathogen genetic sequences into a database, making them available for such analyses. These reporting delays severely decrease precision of phylodynamic methods closer to present time, and for some models can lead to extreme biases. Here we present a method that affords reliable estimation of the effective population size trajectory closer to the time of data collection, allowing for policy decisions to be based on more recent data. Our work uses readily available historic times between sampling and reporting of sequenced samples for a population of interest, and incorporates this information into the sampling model to mitigate the effects of reporting delay in real-time analyses. We illustrate our methodology on simulated data and on SARS-CoV-2 sequences collected in the state of Washington in 2021.

## Author summary

Estimating the number of individuals infected by a given virus is key for informing dynamic health policy, but it is also a nontrivial task. Reported case data often suffers from sampling biases, preventing accurate inference for a population of interest. Pathogen genetic data provide an alternative data source that can be used in phylodynamic analyses that are more robust to sampling biases. Unfortunately, the time between when a sample is collected and when it is sequenced and available for analysis, which we refer to as the reporting delay, results in unobserved samples near present time for real-time analyses. Missing data can be particularly problematic in methods that model the relationship between the number of samples collected over time and the number of infections. Specifically, the concern for those models is that fewer reported samples near present time would result in lower estimates of the true disease prevalence. We propose a model that incorporates information about recent reporting delays to account for missing samples near present time due to having not been reported by the time of analysis. Using simulated data and SARS-CoV-2 sequences from the state of Washington in 2021, we show that our new method ourperforms state-of-the-art methods.

## 1. Introduction

The COVID-19 pandemic demonstrated that fast and accurate analysis of continually collected infectious disease surveillance data is crucial for situational awareness and policy making [1, 2]. Phylodynamic methods form an important set of tools that use genetic sequences of a pathogen of interest to infer its phylogeny and parameters of disease dynamics, such as the effective population size. The effective population size is a measure of genetic diversity, and estimation of effective population size is often of interest because under certain conditions this quantity can be connected to the number of infections in the population [3] or in some cases more directly to transmission [4]. Inference of the effective population size can also be useful to compare the growth of different viral lineages [5, 6], as one part of an argument for the effectiveness of an intervention [7], and ultimately, for informed health policy decisions [8].

The COVID-19 pandemic resulted in a massive push towards sharing sampled pathogenic sequences in public databases such as: GISAID (http://www.gisaid.org/www.gisaid.org), NCBI (http://www.ncbi.nlm.nih.gov/www.ncbi.nlm.nih.gov), and ViPR (http://www.viprbrc.org/www.viprbrc.org). Unfortunately, collected samples can take weeks or even months to sequence, upload to a database, and become available for analysis [9]. We refer to this time between sample collection and sequence reporting as the *reporting delay* for a sample. Reporting delays result in missing data near present time since recently collected samples are less likely to have been sequenced and uploaded yet. During the COVID-19 pandemic, reporting delays were a novel and important consideration to most, with the emerging need for real-time analysis, i.e., analysis conducted up to present time [9]. The distribution of delays can be location, time, and even lineage specific [10], influenced by factors such as sequencing cost and laboratory limited capacity. Researchers who had considered reporting delays for surveillance data in real-time analyses, were limited to methods that utilized only aggregated level reporting delay information [11]. The shared public databases of pathogenic sequences provide a new opportunity to utilize detailed sequence-level data of reporting delays.

Modern methods to estimate effective population size changes from genetic data have evolved from the original coalescent skyline plot where the effective population size trajectory, *N*_*e*_(*t*), was modeled nonparametrically as piecewise constant [12], to grouping methods that resulted in smoother estimates [13], to the first Bayesian coalescent skyline plot model [14] which jointly inferred a pathogen’s evolutionary tree and *N*_*e*_(*t*). Several advancements on the Bayesian coalescent skyline plot models have been proposed in recent years which consider different interval specifications for the piecewise *N*_*e*_(*t*) or regularization methods for *N*_*e*_(*t*). See Ho and Shapiro (2011) and Billenstein and Höhna (2024) for a detailed comparison of Bayesian nonparametric inference of *N*_*e*_(*t*) methods [15, 16]. When pathogen samples are being continually collected over time it is often the case that the frequency at which samples are collected is related to the burden of the infection in the population. This is known as preferential sampling, and Karcher *et al*. (2016) proposed a phylodynamic model that built on Bayesian coalescent skyline plot models to relate the sampling intensity to the effective population size [17]. It was shown that when preferential sampling is present, not accounting for it can result in biases, while accounting for it can result in more accurate and precise inference of the effective population size trajectory. This model has been extended to allow for additional factors to be related to the sampling intensity and effective population size [18, 19].

In this work we use simulations to investigate the effects of reporting delays in real-time phylodynamic inference of the effective population size; we compare the effects across various state-of-the-art inferential strategies. We also propose a strategy to mitigate the effects of reporting delays within the preferential model, by incorporating information about the distribution of recent reporting delays. This extends the Karcher *et al*. (2020) model by including reporting probabilities into the sampling intensity model [18]. We use simulations to compare the performance of our proposed model with competitive real-time phylodynamic strategies in the presence of preferential sampling and reporting delays and show that our model has lower bias, better coverage, and higher precision than state-of-the-art methods. Finally, we use SARS-CoV-2 sequences from Washington state as a case study to compare real-time inferential strategies on data which suffers from reporting delays to the performance of retrospective inference on all sampled sequences in the hypothetical case of no reporting delays.

## 2. Methods

We will begin with a description of the nonparametric phylodynamic methodology proposed in Karcher *et al*. (2020) [18]. This Bayesian strategy will be described starting with how the pathogen genetic samples are modeled conditionally on its evolutionary tree, sampling times, number of samples at each time, and effective population size trajectory, followed by details of the overall full hierarchical model. Once this framework is understood, we will introduce our proposal to mitigate the effects of delays between collecting a sample and depositing a pathogen sequence obtained from the sample into a public database.

### 2.1. Summary of Bayesian nonparametric *N*_*e*_(*t*) inference

When analyzing pathogen evolution, we use an alignment of sampled pathogen genetic sequences as data. These sequences can either be collected at the same time, isochronous sampling, or at different points in time, heterochronous sampling. Here we are concerned with viruses that evolve rapidly with continuously collected samples so we will consider heterochronous sampling of DNA or RNA sequences aligned and stored in matrix $y=\{y_{ji}\}$, j=1,...,n, i=1,...,L, where *n* is the number of sequences and *L* is the alignment length. The sequences, y, all ultimately share a common ancestry, and the evolution of the sequences from their most recent common ancestor is described by a bifurcating tree called a genealogy, denoted as g.

We assume that given the genealogy, alignment sites are independent and identically distributed. The evolutionary changes in the nucleotides present at each alignment site, column of matrix y, are modeled by a continuous-time Markov chain substitution model parameterized by vector θ. From a given viral genealogy and substitution rate matrix, the probability of observing sequences y, P(y|g,θ) can be calculated using an efficient dynamic programming algorithm [20]. Equipped with a model for the alignment, a model is needed for the pathogen’s genealogy.

**Fig 1:**
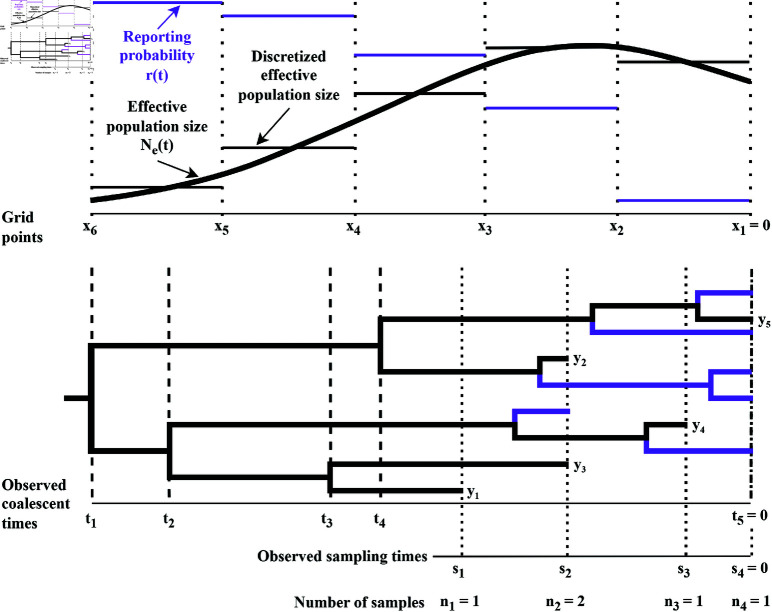
Example of an effective population size trajectory (top figure) and corresponding genealogy (bottom figure), with reporting probabilities and corresponding unobserved tips denoted by purple coloring. For a real-time analysis the reporting probability decreases as the collection date gets closer to present time, time zero.

The lower half of Fig 1 displays a genealogy relating five sequences, black tree tips, collected across four sampling times. Note the purple tips denote samples collected but not yet reported and available for use by the time of analysis. Sampling times are denoted by $s=\{s_{j}{\}}_{j=1}^{m}$ and sample sizes by $n=\{n_{j}{\}}_{j=1}^{m}$ with $n=\sum_{j=1}^{m}n_{j}$. In this set up we imagine generating the genealogy backwards in time starting from the most recent sampling time, *s*_*m*_ = 0. The branches of this evolutionary tree end at the sampling times s, and the convergence, or coalescence, of two branches corresponds to a common ancestor of the two sequences. The tree’s branches coalesce until the most recent common ancestor of all of the samples, the root of the tree. The times of the coalescent events are denoted $\{t_{i}{\}}_{i=1}^{n-1}$, with $t_{1}>...>t_{n-1}$, and the most recent sampling time *s*_*m*_ = 0 will be denoted by *t*_*n*_ because this notation will make it convenient to define the joint density of coalescent times later.

The effective population size, denoted *N*_*e*_(*t*), is a time-varying measure of genetic diversity. The number of active lineages at a time *t* is the difference between the number of sampling and coalescent events between times 0 and *t*. The intervals *I*_*i*,*k*_ are defined by the sampling and coalescent times, so the number of active lineages, denoted *l*_*i*,*k*_, in an interval is constant. For k=2,...,n, the intervals that end in a coalescent event are denoted $I_{0,k}=(max\{t_{k},s_{j}\},t_{k-1}]$, for $s_{j}<t_{k-1}$, and intervals that end with a sampling event are denoted $I_{i,k}=(max\{t_{k},s_{j+1}\},s_{j+i-1}]$, for $t_{k}<s_{j+i-1}\leq s_{j}<t_{k-1}$ with *i*>0. For a more detailed explanation of the *I*_*i*,*k*_ intervals, and a clarifying visual, see Lan *et al*. (2015) [21].

Coalescent models are continuous-time Markov chains used to model a genealogy from a sample of sequences [22]. Rodrigo *et al*. (1999) extended coalescent theory for heterochronous sampling to calculate the joint distribution of a genealogy given its sampling times, number of samples collected at each time, and the effective population size, as the product of conditional densities and tail probabilities of coalescent times [23]:

$$P(g|s,n,N_{e}(t))=\prod_{k=2}^{n}P(t_{k-1}|t_{k},s,N_{e}(t))\;=\prod_{k=2}^{n}\frac{A_{0,k}}{N_{e}(t_{k-1})}\exp\{-\int_{I_{0,k}}\frac{A_{0,k}}{N_{e}(t)}dt-\sum_{i\geq 1}\int_{I_{i,k}}\frac{A_{i,k}}{N_{e}(t)}dt\},$$ (1)

with the coalescent factors Ai,k=(li,k2).

Assuming the effective population size trajectory *N*_*e*_(*t*) is an unknown function in continuous time, the integral in Eq 1 is intractable. We adopt a common approach [24–26], well described by Lan *et al*. (2015), that discretizes the effective population size to be piecewise constant, with changes occurring at evenly spaced time points that form a regular grid $x=\{x_{d}{\}}_{d=1}^{D}$, spanning from the most recent sampling time, $s_{m}=x_{1}$, to the first coalescent time, $t_{1}=x_{D}$ [21]. In this approach we define $N_{e}(t)=\exp[\gamma (t)]$, and approximate *N*_*e*_(*t*) by $N_{e}^{\gamma }(t)=\sum_{d=1}^{D-1}\exp(\gamma_{d})1_{t\in (x_{d},x_{d+1}]}$. The $\gamma_{d}$’s *a priori* follow a first order random walk: $\gamma_{d}|\gamma_{d-1}\sim N(\gamma_{d-1},1/\kappa )$ with $\gamma_{1}\sim N(0,\sigma_{\gamma }^{2})$. We adopt the common approach of using a gamma prior distribution for the hyperparameter κ.

With heterochronous sampling, it is likely that the frequency of sampling is related to the number of infections in the population (e.g., increased sampling intensity when there is an increase in infections). Additional factors may also influence the sampling intensity, such as time variable cost of sequencing a pathogen genome. We denote these additional factors as $f_{2},...,f_{m}(t)$. In the preferential sampling model, we model sampling events as a Poisson Process with intensity λ(t) that depends on such time-varying factors:

$$\log\lambda (t)=\beta_{0}+\beta_{1}\log[N_{e}(t)]+\beta_{2}f_{2}(t)+...+\beta_{p}f_{p}(t).$$ (2)

Note the sampling intensity can include interactions between the covariates and the log effective population size, but we do not include them in our model here. The coefficients $\beta =(\beta_{0},\beta_{1},...,\beta_{p})$’s are assigned independent normal priors with means $\mu_{\beta }$ and variances $\sigma_{\beta }$. Since the effective population size is piecewise constant on the regular grid x, for simplicity we require time-varying covariates also be piecewise constant on the same grid.

Altogether, the posterior we are interested in is

Pr(g,γ,κ,β,θ|y,s)∝Pr(y|g,θ)Pr(g|γ,s)Pr(s|γ,β)Pr(γ|κ)Pr(θ)Pr(κ)Pr(β). (3)

Approximation of this posterior via Markov Chain Monte Carlo (MCMC) is implemented in the phylodynamic software BEAST [18, 27]. This Bayesian inference is time and memory intensive though, so it is common in practice to estimate the genealogy first and assume the genealogy is known. When the genealogy is known the posterior of interest reduces to

Pr(γ,κ,β|g,s)∝Pr(g|γ,s)Pr(s|γ,β)Pr(γ|κ)Pr(κ)Pr(β). (4)

Approximations of this posterior via MCMC and via Integrated Nested Laplace Approximations (INLA) [24] are implemented in the phylodynamic R package phylodyn [28].

### 2.2. Accounting for reporting delays

The time delay between collecting a sample and depositing that sample’s sequence into a database arose as a problem during the SARS-CoV-2 pandemic, because of the urgent need for up-to-date understanding of disease dynamics. Missing the most recent data is especially problematic for the preferential sampling model because of the dependency between the sampling intensity and the effective population size. Intuitively, a model that takes into account preferential sampling would underestimate the effective population size close to the present time due to the lack of observed samples. One possible solution of this problem is to use a coalescent model without the preferential sampling component, avoiding the dependency between the sampling intensity and the effective population size. While the biases from the missing data would be avoided with this strategy, unaccounted preferential sampling can result in biases, and wider credible intervals than those modeled with preferential sampling [17].

Another way to circumvent this missing data issue is to only use data up to a time when all of the data is likely to have been reported (e.g., data up to two months prior to time of analysis). For example, phylodynamics was used to compare SARS-CoV-2 lineages in England with data truncated by two weeks to avoid reporting delays in 2021 [6]. The major pitfall of this truncation strategy is the inability to perform real-time phylodynamics to inform outbreak mitigation, a problem that increases for locations or time periods with extensive reporting delays.

### 2.3. Incorporating reporting delay distribution into preferential sampling model

To mitigate effects of reporting delays on real-time phylodynamic analyses with preferential sampling, we propose incorporating information about the distribution of recent delays in the sampling intensity model. In the preferential sampling model sampling times are modeled as a Poisson process with intensity λ(t). Let r(t) be the probability that a sample collected at time *t* was sequenced and reported by the time of the analysis. Define the observed sampling times, $\tilde{s}$ to be the subset of the true sampling times, s, that are reported by the time of analysis. Then the observed sampling intensity, $\tilde{\lambda }(t)$, could be expressed as the product of the true sampling intensity and the probability of a sample being reported, resulting in a thinned Poisson process with intensity $\tilde{\lambda }(t)=\lambda (t)r(t)$. Plugging Eq 2 into the definition of $\tilde{\lambda }(t)$, we get the following new model for the log-sampling intensity

$$\log\tilde{\lambda }(t)=\log[r(t)]+\beta_{0}+\beta_{1}\log[N_{e}(t)]+\beta_{2}f_{2}(t)+...+\beta_{p}f_{p}(t).$$ (5)

We refer to this proposed adjustment to the preferential sampling model as the delay-aware BNPR PS model.

### 2.4. Implementation of delay-aware BNPR PS model

Our proposed model assumes that the reporting probabilities are known, but in reality they would likely not be known, and they could be changing over time. Reporting and sampling dates are readily available metadata for pathogen sequences, so the user of our method is advised to use these reported metadata for a pathogen of interest to calculate the empirical cumulative distribution function (cdf) of the delays to approximate the reporting probabilities $r=(r_{1},r_{2},...,r_{D})$. Similarly to the effective population size and any covariates, the reporting probabilities r must also be defined as piecewise constant across the regular grid $x=\{x_{d}{\}}_{d=1}^{D}$. When approximating the reporting probabilities there are several considerations: Is there recent data that can provide information about reporting delay behavior? Should the reporting delays of recently reported sequence or recently sampled sequences be used? What should be considered recent?

Our proposed method is not applicable to situations without some data about recent reporting delays, such as the first few weeks of an outbreak where sequencing efforts are just beginning. In a situation with available data on recent reporting delays, it is important to recognize that the empirical cdf of sequences sampled during a predefined time window will provide biased estimates of reporting probabilities due to unreported sequences being censored. In the scenario with all sampling times known, regardless of sequences being reported by the time of analysis, survival analysis methods (e.g., Kaplan-Meier estimator) could be used to approximate the reporting probabilities. Otherwise, the empirical cdf of samples reported during a predefined time window should be used to estimate reporting probabilities.

When considering what time frame of reported sequences to use, it is important to look at the most recent reporting delays and examine if the delay behavior is changing overtime. The time frame of reported sequences to use should be chosen from the time of analysis back to a time where the reporting delay behavior continues to be relatively consistent. This is important because more data can provide better estimates, but reporting delays from sequences collected during a time period with very different delay behavior will not approximate current delay behavior well. Further investigation of these modeling decisions and their impact on the inference with the delay aware BNPR PS model is provided in S1 Appendix Sect 3.

We developed a new version of the R package **phylodyn** [28], **phylodyn2** (https://github.com/CatalinaMedina/phylodyn2), which has a well-documented subset of the functionality of phylodyn, with the additional ability to account for reporting delays in real-time analyses through our proposed delay-aware BNPR PS model. The R package phylodyn included several posterior sampling strategies. For phylodyn2 we chose to focus on the Integrated Nested Laplace Approximations (INLA) based strategy to approximate the marginal posterior distributions $Pr(\gamma_{i}|g)$ because it is faster than the MCMC-based method. This INLA implementation formulates the model for sampling times as a Poisson regression [24]. A helpful observation is that the log[r(t)] term in Eq 5 could be operationally viewed as an offset to this Poisson regression. However, the original phylodyn implementation did not allow for inclusion of a user specified offset term. That is one major change present in phylodyn2 that allowed for the implementation of the delay-aware BNPR PS model, where the reporting probabilities are calculated from a user specified vector of recent reporting delays.

Another way of viewing the log[r(t)] term within the framework developed in [18] is considering it to be a time-varying covariate of the sampling intensity, with a coefficient of the value one. The appeal of this perspective is the ease of implementation with existing phylodynamic tools that allow for time-varying covariates in the sampling intensity, such as BEAST and phylodyn. One could specify log[r(t)] as a regression covariate with a narrow prior for the coefficient of this term centered at one. This adds unnecessary randomness, since the coefficient of this term is theoretically one, but the ease of use makes this option worth exploring. This implementation is also available in phylodyn2, and its performance is examined in S1 Appendix Sect 1.1.

All code to reproduce the results in this paper can be found at https://github.com/CatalinaMedina/reporting-delays-in-phylodynamics-paper.

## 3. Results

### 3.1. Simulations

We performed simulation studies to mimic real-time phylodynamic analyses in the presence of preferential sampling, aiming at two primary objectives. Firstly, to investigate the effects of reporting delays with currently available phylodynamic inferential strategies. Secondly, to compare the performance of our proposed model against the currently available strategies. Of key interest is how well the effective population size trajectory can be inferred close to the most recent sampling time.

Three real-time inferential strategies were considered for comparison: avoid modeling the sampling time dependency by using the the Bayesian nonparametric phylodynamic reconstruction (BNPR) model, model the sampling time dependency with the Bayesian nonparametric phylodynamic reconstruction with preferential sampling (BNPR PS) model, and model the sampling time dependency and reporting delays with our proposed delay-aware BNPR PS model. We also fit the BNPR PS model to all of the data, regardless of whether it was reported, to provide a retrospective baseline for the performance of these real-time inferential strategies.

**Fig 2:**
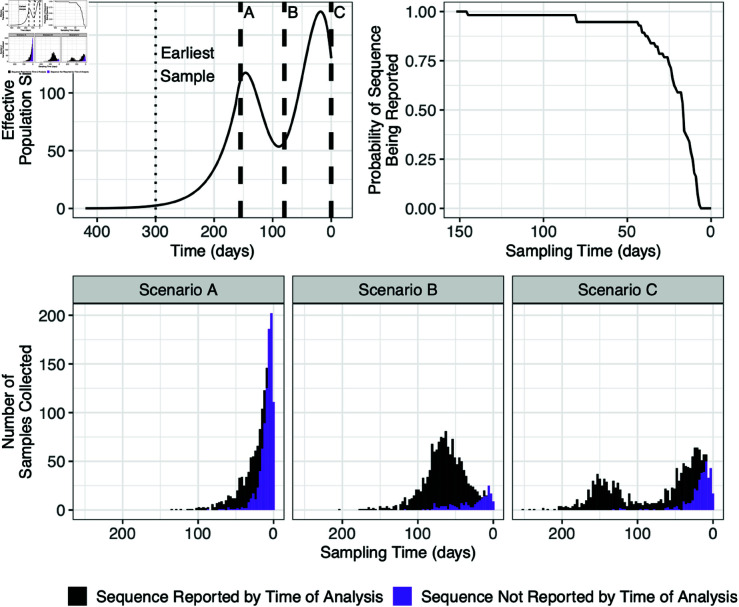
Simulation details: effective population trajectories (upper left plot), reporting probability by sampling time (upper right plot) obtained from the Washington state data, and histograms of sampling times from the last simulation of in each simulation scenario, approximately 1500 samples each, colored by whether sample was reported by time of analysis (bottom plots). Each simulation scenario had a different time zero, i.e., time of latest sample (dashed lines). The earliest sampling time in each scenario was at the same point in the trajectory (dotted line).

We used three simulation scenarios with the same effective population size trajectory, but across different time periods so that the effects of reporting delays with different trajectory behavior near time zero could be investigated. The upper-left panel of Fig 2 shows the effective population size trajectory, as well as the most recent sampling time, the time of analysis, for each scenario. Since time is viewed in reverse, the most recent sample in simulation scenario C is time zero, and the earliest sample was 300 days prior. Scenarios A and B had sampling time periods of 150 days and 220 days, respectively. Scenario A is meant to resemble an initial outbreak, which would have fewest samples due to reporting delays. Scenario B allows us to examine behavior when there is a slight increase occurring near present time, but less with sampling. Lastly, in scenario C there is a decline near present time and the recent peak corresponds to more reported samples near time zero than in scenario B. In both scenarios B and C the recent change in trajectory direction would be expected to be difficult to detect due to reporting delays.

Sampling times were simulated from an inhomogeneous Poisson process with intensity $\lambda (t)=\exp(\beta_{0})[N_{e}(t){]}^{\beta_{1}}$. Coalescent times were simulated using the coalsim() function in the phylodyn R package [28], which uses a time-transformation technique where the coalescent likelihood is treated as an inhomogeneous Poisson process [29]. Parameter $\beta_{1}$ was set to 2 to create a reasonably strong preferential sampling effect and $\exp(\beta_{0})$ was selected to achieve a sample size of approximately 1500 samples, each with its own sampling time.

For each sampling time we simulated a random Bernoulli to indicate if a sample was reported by the time of analysis. Sampling times for each scenario are plotted in Fig 2 and colored by whether it was observed or not. To create realistic delays, the reporting probabilities were obtained from the empirical reporting delay distribution of SARS-CoV-2 sequences collected in the state of Washington. See the real data investigation results subsection for details, visualized in upper-right panel of Fig 2. The tips of the genealogy of the full tree that correspond to unreported samples were pruned from the tree, to get the observed genealogy. Each inference was performed with the INLA-based Bayesian phylodynamic inference implemented in the R package phylodyn2.

**Fig 3:**
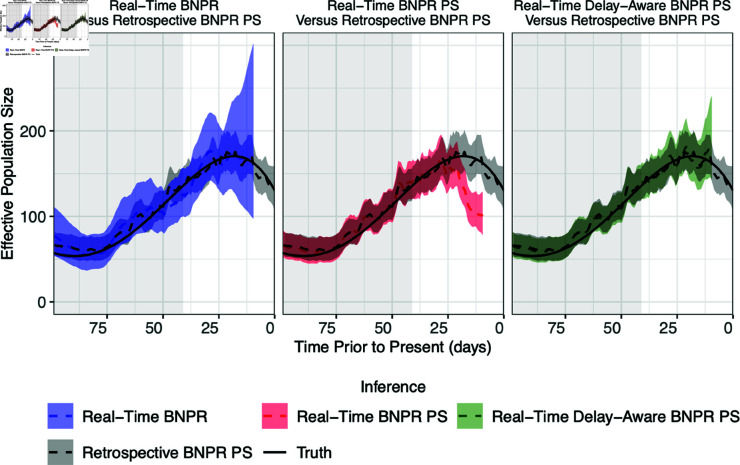
Comparison of real-time phylodynamic methods to infer the effective population size trajectory for a single simulation in scenario C, with reporting delays and preferential sampling present. Median estimates of the effective population size and 95% credible intervals are plotted. The BNPR PS model was also applied retrospectively with all of the sampled sequences, regardless of if they were reported by time zero, to serve as a reference for comparison for the three real-time inference methods. The white background indicates the recent time period likely suffering from reporting delays, specifically where reporting probabilities (RPs) are below 90%, and is therefore the region of interest.

We will begin by discussing the results of a single simulation within a scenario, in order to better understand the patterns in the performance of each inference strategy across all of the simulations. Plots of the results of each inference strategy from a single simulation in each simulation scenario are available in S1 Appendix Sect 1.1. Fig 3 plots the true (solid lines) and inferred (dashed lines) effective population size trajectory for the 100 days prior to the most recently collected sample. Here we focus on the two options of real-time inference, the BNPR and BNPR PS models, and our proposed delay-aware BNPR PS model. While the ultimate goal is to be able to infer the true effective population size trajectory, it is useful to see how closely the data generating model can approximate the true trajectory, within simulation scenario C, where the trajectory of interest is on the decline at present time. This is why each plot also contains the BNPR PS inference performed retrospectively on all of the data, not just the observed data – this serves as a baseline to compare the inference of *N*_*e*_(*t*) from each real-time inferential method. The white background indicates the time period of interest, where delays are probable, and conversely the gray background indicates the period where reporting delays are unlikely. We chose to use the 90th percentile of the Washington state data reporting delays distribution, which was 41 days in this case, as the cutoff for these two periods.

In the first panel of Fig 3 we see real-time inference with the BNPR model, which ignores the dependency between *N*_*e*_(*t*) and the sampling time. The BNPR model appears to have relatively low bias, but wide 95% credible intervals that increase in width near time zero. The real-time inference with the BNPR PS model stands out because of the bias which increases as time approaches the most recently collected sample. This demonstrates the bias introduced when using the data generating model, the BNPR PS model, when there are reporting delays present in the data. Alternatively, our implementation of the delay-aware BNPR PS model has less bias than the BNPR PS model near time zero and visibly narrower 95% credible intervals than the BNPR model near time zero.

**Fig 4:**
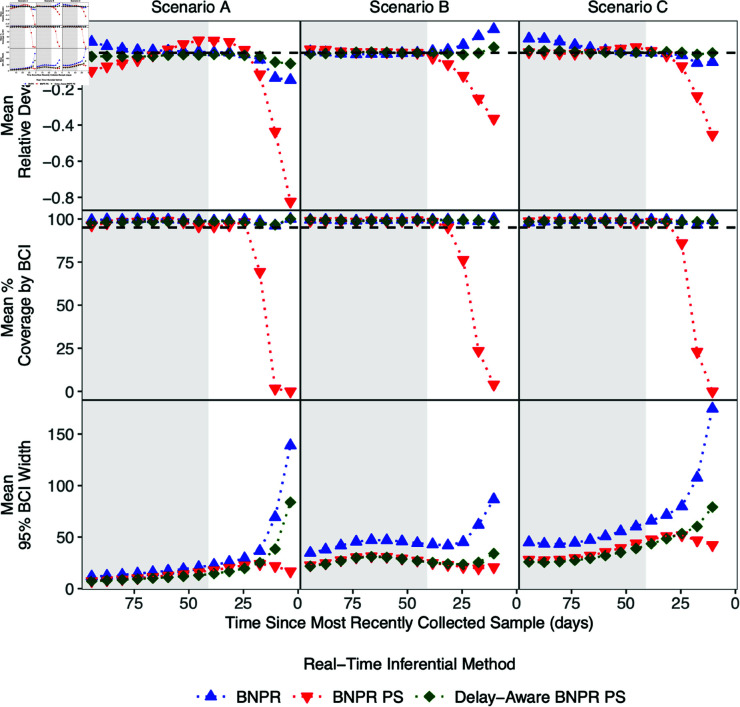
Seven-day moving averages of the mean relative deviation, mean percent coverage, and mean interval width by the 95% Bayesian credible intervals, for each real-time phylodynamic strategies to infer the effective population size in each simulation scenario with preferential sampling (PS) and reporting delays in the observed data. Real-time inference was performed with the Bayesian nonparametric phylodynamic reconstruction (BNPR) model, BNPR PS model, and with the delay-aware BNPR PS model with 500 simulations per scenario.

The results identified from the single simulation in Fig 3 generally persist across all 500 simulations, in each of the three simulation scenarios, visualized in Fig 4. The plots present a seven-day moving average of the mean relative deviation, mean percent of 95% Bayesian credible intervals which covered the true value, and mean 95% credible interval width for each inference strategy in each simulation scenario. A moving average was chosen because a metric of the inference over the entire time period would be insufficient to describe how inference performance changes with proximity to time zero. Mean relative deviation is the most important of the three chosen metrics because it assess accuracy, of the point estimate, interval coverage was selected to examine the accuracy of the uncertainty of the estimates, and interval width was useful for assessing precision to compare those models with good accuracy and good coverage. Since the performance of these estimation strategies near time zero is of key interest, these plots were truncated to the most recent 100 days. To view the performance metrics results for all inferential methods considered see S1 Appendix Tables A-C.

Focusing on the time period of interest, the most recent 41 days, our proposed delay-aware BNPR PS model consistently has lower mean relative deviation than the BNPR method, in each seven-day moving average, though there is not much practical difference. The BNPR PS model has increasing relatively large mean relative deviation as sampling times decrease to time zero, in each simulation scenario. The absolute maximum mean relative deviations in scenario A are all achieved in the week prior to time zero are 0.15, 0.82, and 0.06 for the BNPR, BNPR PS, and our delay-aware BNPR PS model respectively. The 95% Bayesian credible intervals for the BNPR and our delay-aware BNPR PS model are consistently conservative, while the BNPR PS model’s 95% credible intervals’ coverage drops below 95% and approaches 0% as sampling time approaches time zero. Finally, while maintaining competitively low bias and high coverage, our proposed model consistently has lower mean 95% Bayesian credible interval widths than the BNPR model, with the difference between the two models increasing as sampling time approaches time zero.

### 3.2. Real data investigation: Washington state COVID dynamics

**Fig 5:**
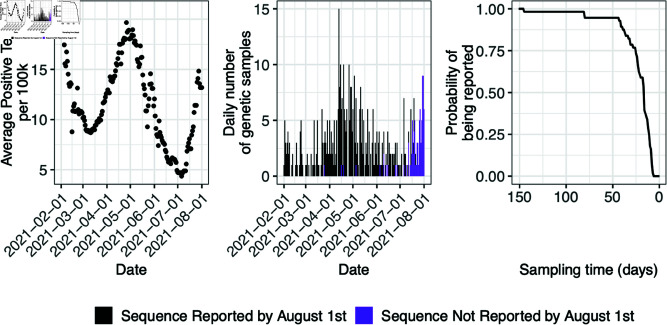
Left plot is the seven-day rolling average of positive COVID-19 tests per 100,000 people in the state of Washington. Middle panel shows number of SARS-CoV-2 genetic samples collected in Washington state, colored by whether the sample was reported by the time of analysis, August 1, 2021. Right panel shows empirical cumulative distribution of reporting delays from the month prior to time of analysis.

We used SARS-CoV-2 sequences from Washington state for the purpose of investigating the differences between a real-time phylodynamic analysis with and without our proposed method to account for reporting delays in genomic data. The SARS-CoV-2 sequences were accessed via the GISAID database available at https://gisaid.org/EPI_SET_220330me, for Washington state sampled between February 01, 2021 and August 01, 2021, inclusive [30]. This time period was of interest because researchers were regularly sequencing Washington samples at this point in the pandemic, and the reporting behavior is relatively consistent during this period. Fig 5 plots seven-day rolling average number of COVID-19 cases per 100,000 people in population in the state of Washington, the daily number of SARS-CoV-2 samples available in GISAID for Washington, colored by whether the sequence was sampled by August 01, 2021 (middle plot), and the empirical cumulative distribution function for sampling dates between July 01, 2021 and August 01, 2021.

The observed data are samples that had been sequenced and reported to GISAID on or before August 01, 2021, time zero of our analysis. The 90th percentile of the reporting delays distribution is 41 days. Since we are interested in the inference of *N*_*e*_(*t*) when reporting delays are present, we chose to focus our attention on the most recent 41 days.

Genealogy estimation was performed in BEAST for each data set: all 500 sequences to represent a retrospective analysis, the observed 412 sequences to represent a real-time analysis, and 375 remaining sequences after truncating any sequence with sampling times larger than 41 days to avoid reporting delays in a near real-time analysis. We used the HKY substitution model with empirically estimated base frequencies [31], Bayesian Skygrid coalescent model [25, 32], and a Uniform prior on the clock rate between $2.38\times 10^{-3}$ and $8\times 10^{-4}$ [33]. The MCMC was run for $25\times 10^{6}$ iterations, logging parameters every 2000th iteration. The maximum clade credibility tree of the posteriors were used as the known genealogy in the phylodynamic reconstruction, for each of the three analyses. See S1 Appendix Sect 2 for more details about this analysis to obtain the genealogies.

**Fig 6:**
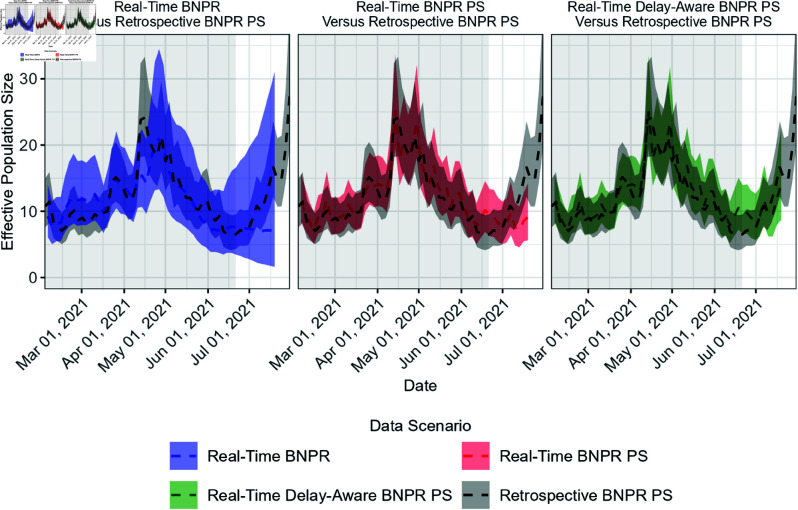
Bayesian nonparametric phylodynamic reconstruction (BNPR) methods used to infer effective population size trajectory for SARS-CoV-19 in Washington state. Each panel shows the inference from a real-time analysis on data suffering from reporting delays and from a retrospective analysis with completely reported data. The white background indicates the recent time period likely suffering from reporting delays.

Inference of the effective population size was performed with the same strategies used in our simulations to compare the performance of our proposed methods against available options. Fig 6 shows the inference of the effective population size for three modeling strategy: Bayesian nonparametric phylodynamic reconstruction (BNPR), BNPR with preferential sampling (BNPR PS), and our proposed delay-aware BNPR PS model.

The retrospective analysis with all of the collected samples with the BNPR PS model infers a peak in transmission activity in mid April of 2021, dropping to a minimum in mid June, followed by a steady increase continuing into August 2021. The results for these analyses are consistent with the trajectory of COVID-19 cases for this time period, visualized in Fig 5, with approximately a two week delay which could be due to reporting delays in COVID test results.

When comparing the real-time analyses we see similar patterns as those identified in our simulations. Using the retrospective BNPR PS model for comparison we see near real time, the BNPR model suffers from low precision, the BNPR PS model’s credible intervals disagree with the retrospective analysis credible intervals, and our proposed delay-aware BNPR PS model is consistent with the retrospective BNPR PS model results, with higher precision than the BNPR model. This gain in precision found with our proposed model would have allowed real-time analysis to infer the increase near present time that the two currently available competitive methods underestimated.

## 4. Discussion

In this work we investigated the effects of reporting delays on real-time phylodynamic methods to infer the effective population size and we proposed the delay-aware BNPR PS model. Through simulations we demonstrated that when preferential sampling is present, real-time analysis with the BNPR PS model suffers from increasingly extreme bias when inferring the effective population size near present time, implying that the BNPR PS model should not be used for such cases because it is unreliable. We also showed that when the preferential sampling relationship is not modeled, real-time analysis with the BNPR model has less bias than the BNPR PS model, but is largely uninformative due to its low precision near present time. Across simulations we found our delay-aware BNPR PS model to perform comparably to the BNPR model in terms of accuracy, without suffering from the same biases as the BNPR PS model. We also found that with more data our model obtains increased precision near present time, relative to the BNPR model. Our results support the intuition that we can infer *N*_*e*_(*t*) more accurately and precisely with more data, specifically when there are more samples sequenced and available for analysis. Beyond the simulations, our Washington data analysis found evidence of preferential sampling and behavior consistent with out simulation results: we saw agreeable results between our delay-aware BNPR PS model and the retrospective BNPR PS model, the real-time BNPR model had very low precision near present time, and the real-time BNPR PS model strongly disagreed with the retrospective BNPR PS model. The simulated and real data results provide compelling arguments that reporting delays should not be ignored in real-time analysis, and that the effective population size trajectory is a reasonable indicator for the effective number of infections.

For simplicity, we assumed the pathogen genealogy is known in our implementation of the delay-aware BNPR PS model in phylodyn2, obtaining the marginal posteriors of *N*_*e*_(*t*) with INLA. The value of this choice is that it is fast and can handle much larger number of sequences than BEAST which jointly infers the genealogy and other model parameters, including *N*_*e*_(*t*). Computational speed and feasibility are necessary considerations with Bayesian phylodynamic methods, especially with online surveillance. It is also important to know how much the unaccounted phylogentic uncertainty from the tree affects our inference. In S1 Appendix Sect 4 we investigated the effect of the phylogenetic tree on the ultimate inference of the effective population size. The results suggest that we are underestimating the effective population size uncertainty, which is expected. A natural next step from this work would be to incorporate our reporting probability adjustment into the joint posterior inferred by the BNPR PS model in BEAST.

Our delay-aware BNPR PS model currently assumes that reporting probabilities are known, and our implementation uses recent reporting delays to estimate current reporting probabilities. This strategy is limited to locations, times, lineages, and even laboratories where there is believed to be consistency in reporting delays for sequences [10]. As such, care is necessary when defining the reporting probability distribution for use in the sampling intensity of our model. We investigated the effects of misspecification of the reporting probabilities, available in S1 Appendix Sect 3, and found the delay-aware BNPR PS model to be preferable to the BNPR PS model, even with relatively major misspecification of the reporting probabilities. That being said, the results do show better performance with better specification of the reporting probabilities. With major misspecification of the reporting probabilities, the BNPR model is preferable because it better reflects the uncertainty of the estimates. The next extension of this work would be to jointly infer the reporting probabilities and the effective population size. This could allow for increased accuracy and better uncertainty quantification, especially for areas with rapid changes in reporting behavior. Perhaps of most interest would be to allow for the reporting delay distribution to change overtime, allowing for updated surveillance of the effective population size with continual data collection.

The BNPR PS method models the sampling intensity parametrically, so naturally there may be concern of model misspecification, especially when studying new variants of unknown infectiousness. Cappello and Palacios (2022) proposed a model which allows for the relationship between the effective population size and the sampling intensity to vary with time as follows: $\lambda (t)=\beta (t)N_{e}(t)$, where β(t) is inferred nonparametrically from the genetic and sampling time data [19]. It would be of interest to extend this model to incorporate known reporting probabilities. The next question would be if it could jointly infer reporting probabilities and *N*_*e*_(*t*) with the time-varying β(t).

Another avenue of interest for future work is to investigate the impact of reporting delays on another class of models. There are two ways to estimate pathogen spread via phylogenies: 1) coalescent and 2) birth-death models. The second class of models needs to model sampling rates by default, which is not true for coalescent-based models, so it would be natural to extend our framework to birth-death models since the observed sampling rates near present time would be impacted by reporting delays [34].

Our proposed delay-aware BNPR PS model is a first step in mitigating the effects of reporting delays on real-time phylodynamic analyses. This work has important implications for real-time research with genomic data. We identified that the data generating model can be biased when ignoring the presence of missing data near present time due to reporting delays. The severity of this bias increases as the number of sequences observed decreases, but this bias can be corrected by using historical data about reporting delays.

## 5. Supporting information

S1 Appendix Additional text, tables, and figure for the manuscript (PDF)
