# Supplementary material for: Accounting for reporting delays in real-time phylodynamic analyses with preferential sampling
Source: PLoS Comput Biol. 2025 May 6;21(5):e1012970. doi: 10.1371/journal.pcbi.1012970 (PMC12101774; doi:10.1371/journal.pcbi.1012970)
Supplement: S1 Appendix — (PDF) [file pcbi.1012970.s001.pdf]

Supplemental materials: Accounting for reporting delays in real-time phylodynamic analyses with preferential sampling

Catalina M. Medina Julia A. Palacios Volodymyr M. Minin

Table of contents

1 Simulation results **2**
1.1 Further simulation results with Washgtinon state's reporting delays 3
1.2 Simulation with Santa Clara County reporting delays (more extreme delays than Washington state) 12

2 Real data investigation: Washinton state COVID dynamics **20**
2.1 Sequences from GISAID 20
2.2 BEAST modeling details for real data investigation: Washington State 22

3 Investigation of reporting probability options **23**
3.1 Options for calculating reporting probabilities 23
3.2 Comparison of inference with simulated data with different historic data options 24
3.3 Inference with Washington data with different historic data options 28

4 Investigation of different phylogenies estimated using Washington data **29**

1 Simulation results

In the manuscript we focused on three real-time inferential strategies: BNPR, BNPR PS, and our proposed delay-aware BNPR PS model with reporting probabilities incorporated as an offset in the model of the sampling intensity. For completeness, we also considered the truncation technique, where only samples collected up until some point in time are used. This is meant to avoid the most recent time period, and therefore avoid reporting delays in data, but by definition is not much of a real-time analysis. Regardless, we investigated this strategy to show that the truncation technique can result in important recent behavior remaining unknown.

We also considered an alternative implementation of our reporting delay-aware BNPR PS model. This alternative implementation is discussed in the last subsection of our methods section in the manuscript. The implementation with reporting probabilities being incorporated as an offset is useful because any existing software that can incorporate a time varying covariate into the log sampling intensity equation would be automatically able to implement our proposed model. As expected, this implementation performed similarly to our originally proposed implementation, with the caveat of increased variation due to the need to model the coefficient of this regression term, which is known to be one in theory.

We also additionally consider the BNPR model used retrospectively on all data. We included this because, while the BNPR PS model is the data generating model in this case, it is less commonly used than the BNPR model. An important finding is that the real-time inference with our proposed reporting delay-aware BNPR PS model on only reported samples is competitive with the retrospective BNPR model fit to all of the data, further supporting why our model should be used.

1.1 Further simulation results with Washgtinon state's reporting delays

1.1.1 Results from last simulation in each scenario

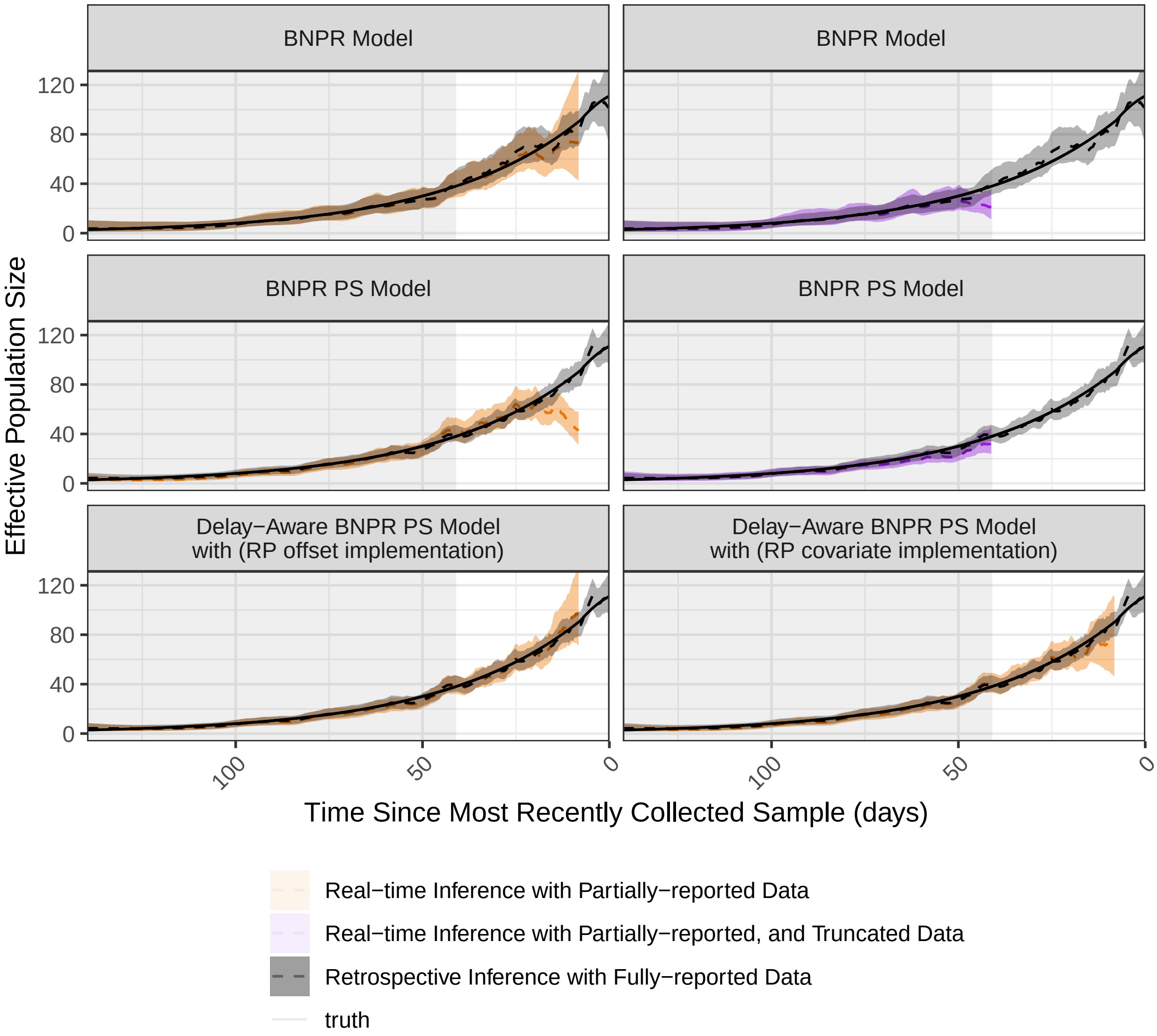

Fig A. Comparison of phylodynamic estimation methods of effective population size trajectory for three different simulated data scenarios from the scenario A trajectory.

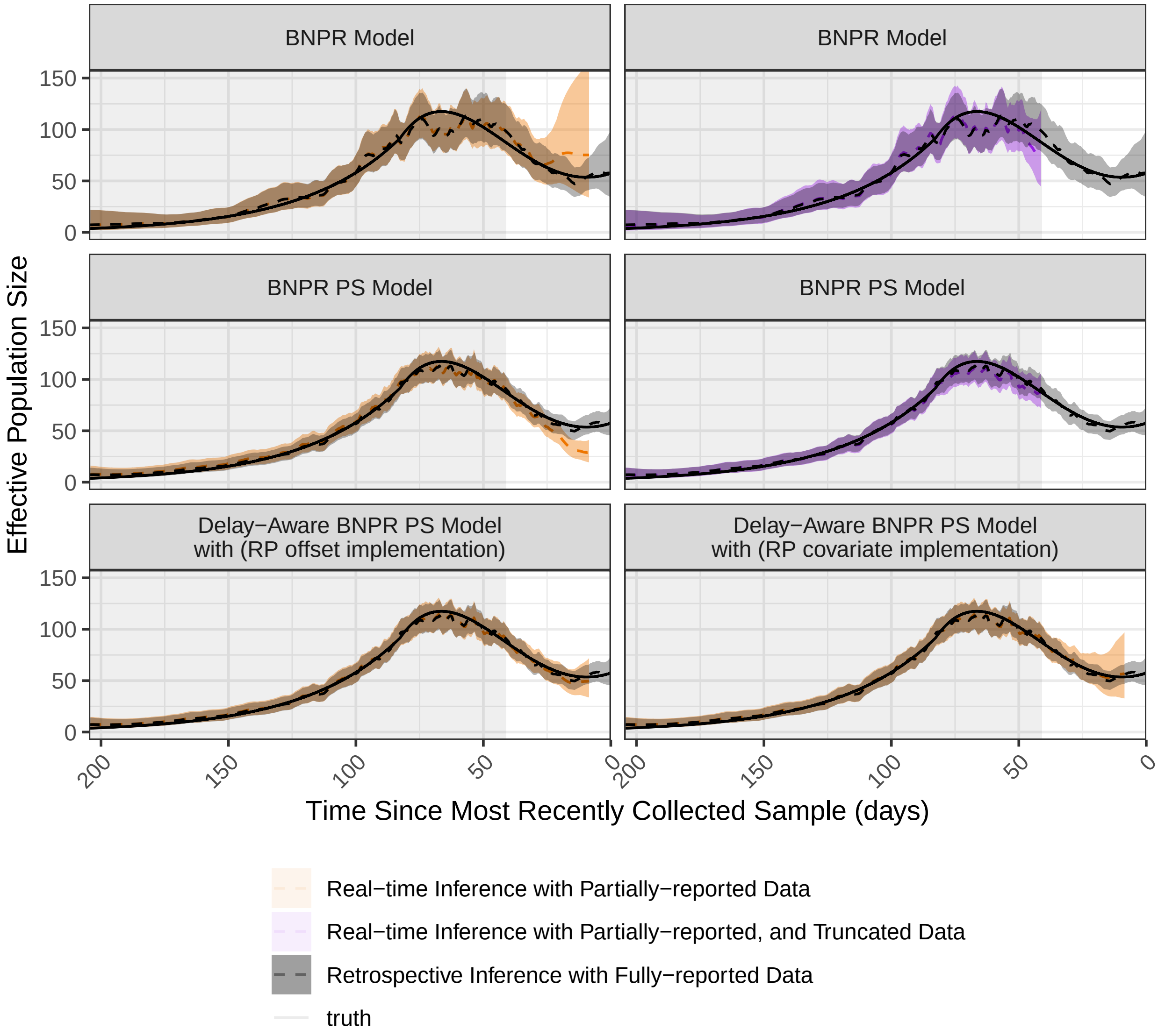

Fig B. Comparison of phylodynamic estimation methods of effective population size trajectory for three different simulated data scenarios from the scenario B trajectory.

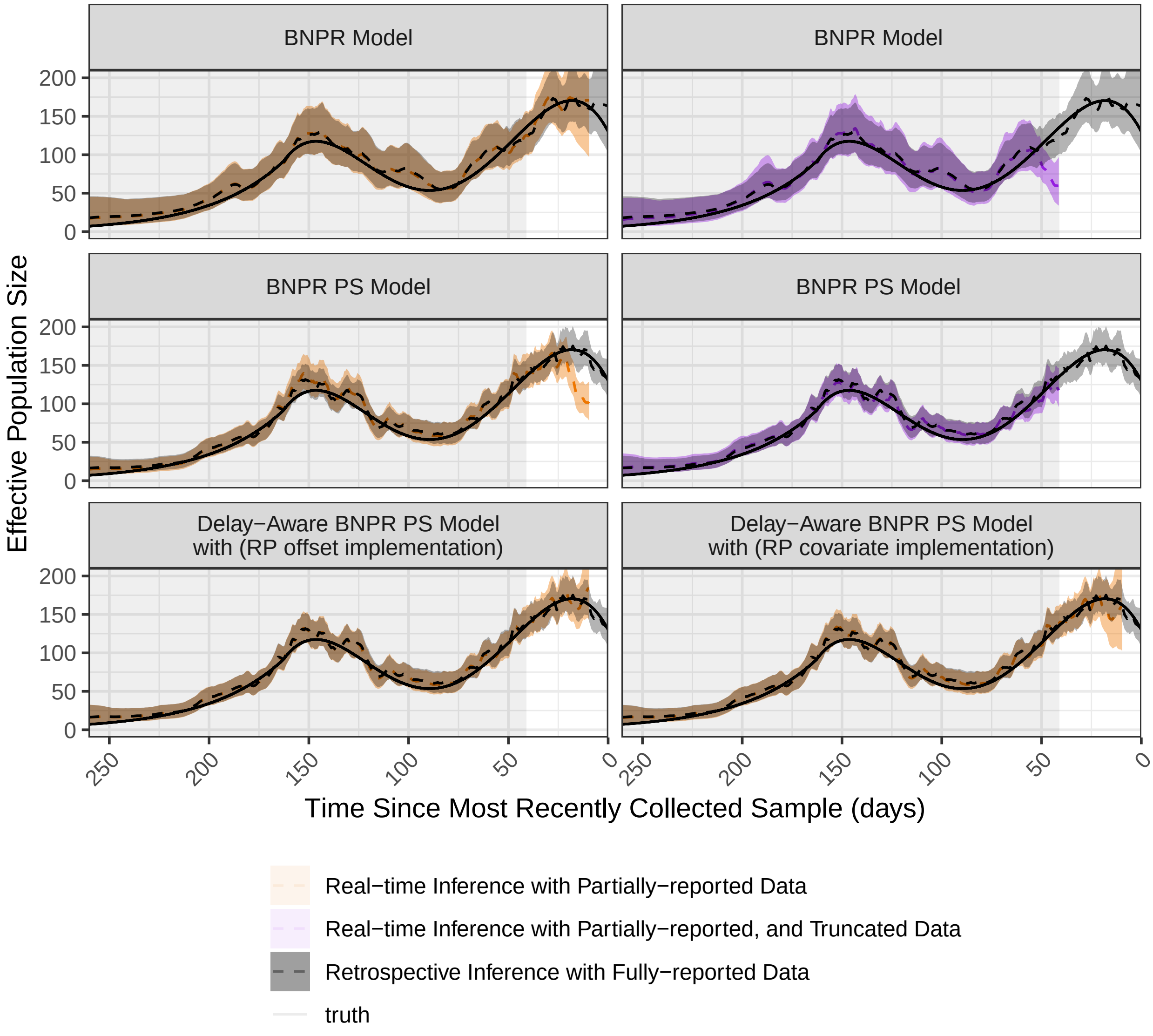

Fig C. Comparison of phylodynamic estimation methods of effective population size trajectory for three different simulated data scenarios from the scenario C trajectory.

1.1.2 Performance metrics across all simulations

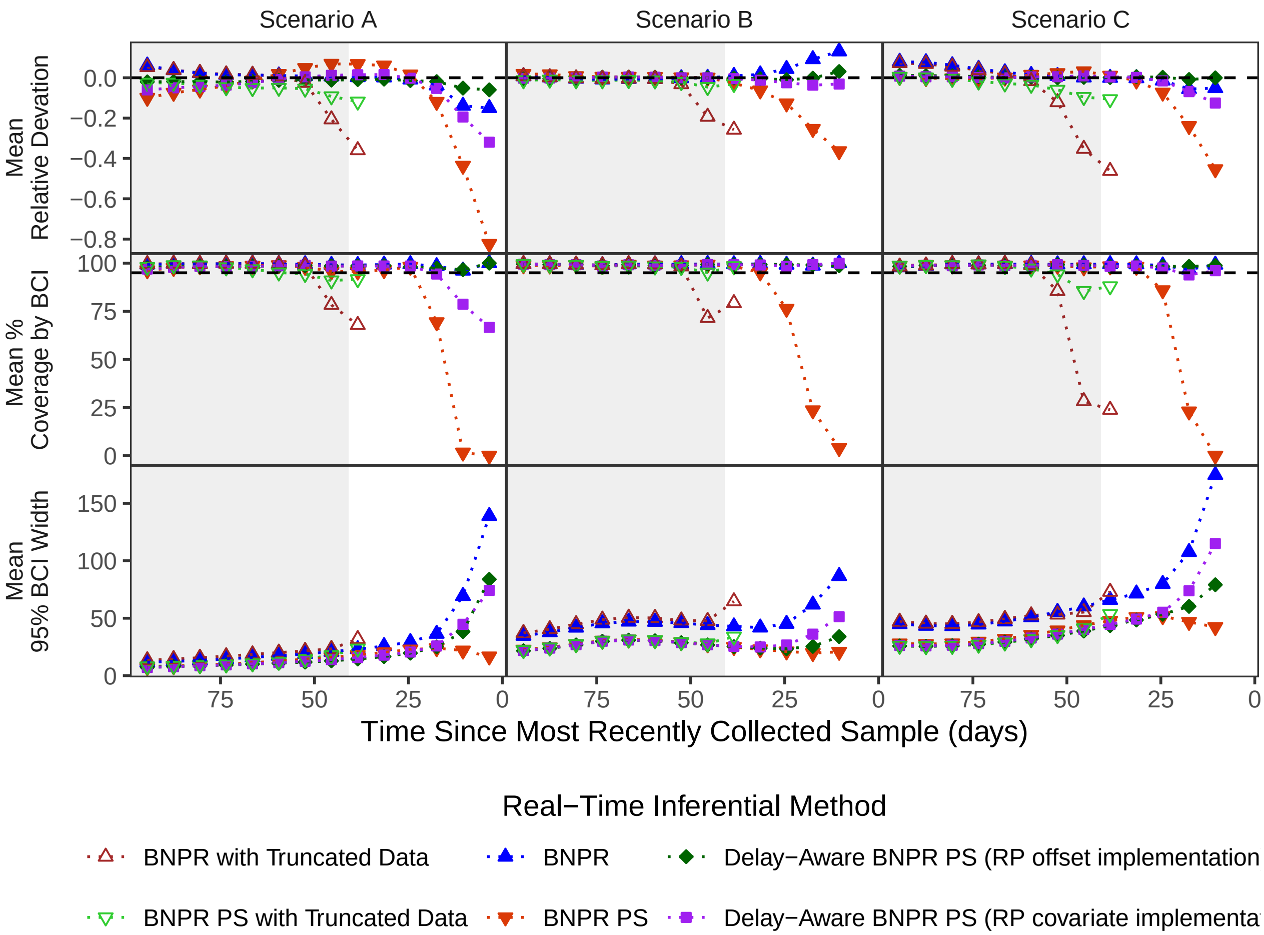

Fig D. Seven-day moving averages of the mean relative deviation, mean percent coverage, and mean interval width by the 95% Bayesian credible intervals, for each real-time phylodynamic strategies to infer the effective population size in each simulation scenario with preferential sampling (PS) and reporting delays in the observed data. Inference was performed with Bayesian nonparametric phylodynamic reconstruction (BNPR), BNPR PS, and delay-aware BNPR PS.

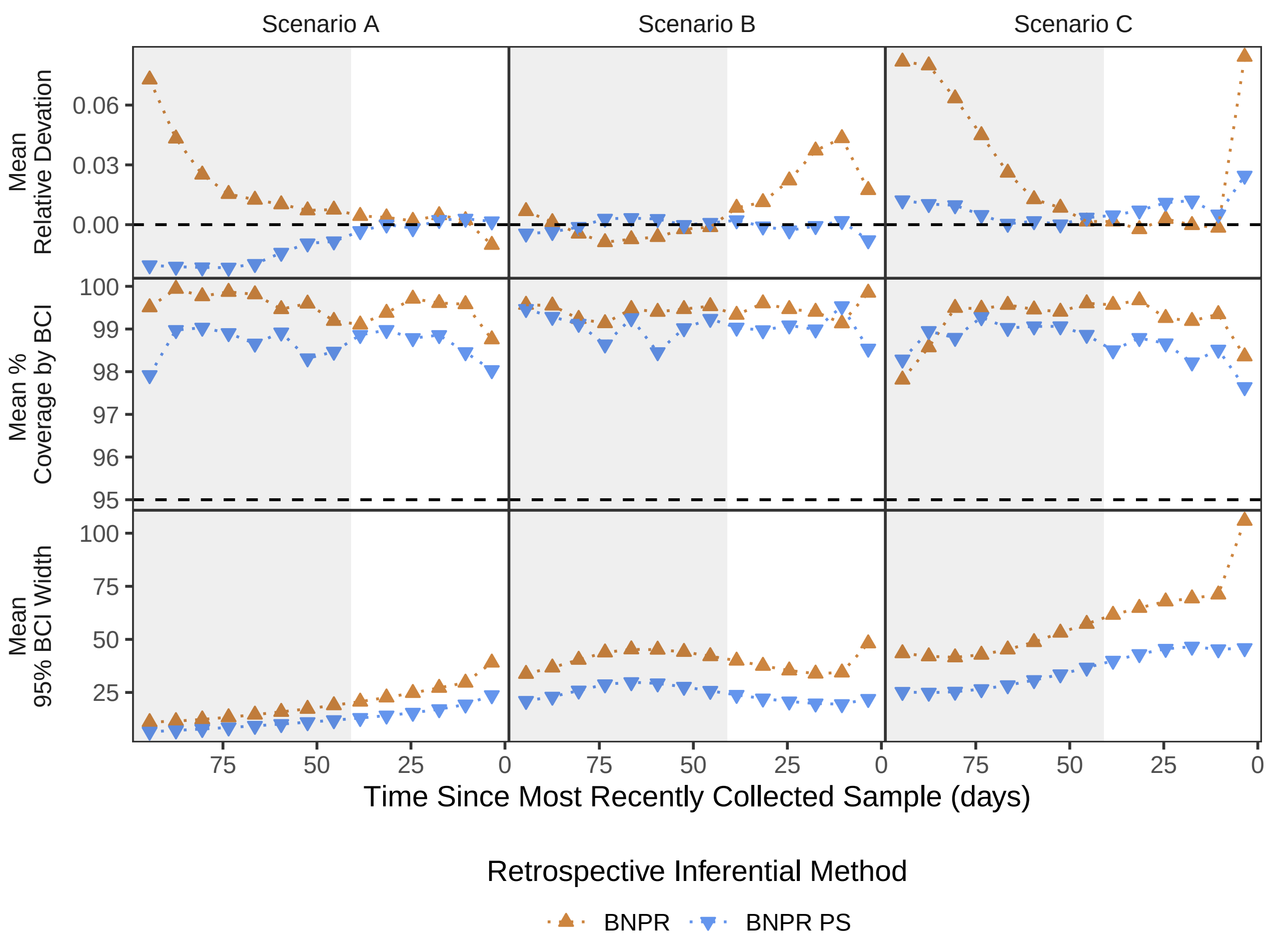

Fig E. Seven-day moving averages of the mean relative deviation, mean percent coverage, and mean interval width by the 95% Bayesian credible intervals, for each retrospective phylodynamic strategy to infer the effective population size in each simulation scenario with preferential sampling (PS) and no reporting delays in the observed data. Inference was performed with Bayesian nonparametric phylodynamic reconstruction (BNPR) and BNPR PS.

Table A. Mean relative deviation of all inferential techniques for effective population size in each data scenario for 500 simulations in scenarios A, B, and C. Time periods span approximately from first simulated sampling time to 90th percentil of historic reporting delays, 41 days. The final time period for each simulation scenario spans from the first to last sampling time simulated.

	Retrospective		Real-Time Inference					
Time Period (days)	BNPR	BNPR PS	Trunc. BNPR	Tunc. BNPR PS	BNPR	BNPR PS	Delay-Aware BNPR PS (RP offset implementation)	BNPR PS (RP covariate implementation)
**Scenario A**								
[0,7)	-0.01	0.00			-0.15	-0.82	-0.06	-0.32
[7,14)	0.00	0.00			-0.14	-0.44	-0.05	-0.19
[14,21)	0.00	0.00			-0.04	-0.12	-0.02	-0.05
[21,28)	0.00	0.00			-0.01	0.02	-0.01	0.00
[28,35)	0.00	0.00			0.00	0.06	-0.01	0.02
[35,42)	0.00	0.00	-0.36	-0.12	0.00	0.07	-0.01	0.01
[0,154]	0.12	0.05	0.10	0.05	0.10	-0.02	0.05	0.01
**Scenario B**								
[7,14)	0.04	0.00			0.13	-0.36	0.03	-0.03
[14,21)	0.04	0.00			0.09	-0.25	0.00	-0.04
[21,28)	0.02	0.00			0.04	-0.13	-0.01	-0.02
[28,35)	0.01	0.00			0.02	-0.06	0.00	-0.01
[35,42)	0.01	0.00	-0.26	-0.03	0.01	-0.03	0.00	0.00
[0,228]	0.14	0.07	0.12	0.06	0.14	0.07	0.06	0.06
**Scenario C**								
[7,14)	0.00	0.01			-0.05	-0.45	0.00	-0.13
[14,21)	0.00	0.01			-0.06	-0.24	-0.01	-0.07
[21,28)	0.00	0.01			-0.01	-0.07	0.00	-0.02
[28,35)	0.00	0.01			0.00	-0.01	0.01	0.00
[35,42)	0.00	0.00	-0.46	-0.11	0.00	0.01	0.00	0.00
[0,307]	0.14	0.08	0.12	0.08	0.13	0.04	0.08	0.06

Table B. Pecent of 95 percent Bayesian credible intervals that covered the true effective population size, from all inferential techniques in each data scenario for 500 simulations in scenarios A, B, and C. Time periods span approximately from first simulated sampling time to 90th percentil of historic reporting delays, 41 days. The final time period for each simulation scenario spans from the first to last sampling time simulated.

Retrospective	Real-Time Inference

Time Period (days)	BNPR	BNPR PS	Trunc. BNPR	Tunc. BNPR PS	BNPR	BNPR PS	Delay-Aware BNPR PS (RP offset implementation)	BNPR PS (RP covariate implementation)
**Scenario A**								
[0,7)	98.76	98.04			100.00	0.00	100.00	66.67
[7,14)	99.58	98.46			96.13	1.62	96.59	78.69
[14,21)	99.61	98.86			98.44	69.31	97.42	94.37
[21,28)	99.71	98.79			99.52	98.14	98.10	98.52
[28,35)	99.38	98.98			99.28	96.37	98.69	98.55
[35,42)	99.10	98.86	67.90	91.65	99.03	95.64	98.58	98.57
[0,154]	98.75	96.79	97.21	95.84	98.71	90.96	96.71	96.32
**Scenario B**								
[7,14)	99.13	99.53			100.00	3.98	98.62	99.95
[14,21)	99.40	98.99			98.75	23.54	99.07	99.24
[21,28)	99.47	99.08			99.19	76.29	99.24	98.40
[28,35)	99.60	98.97			99.62	95.20	99.25	99.08
[35,42)	99.33	99.03	79.24	98.40	99.45	98.39	99.19	99.18
[0,228]	98.06	96.21	97.14	96.20	98.21	89.79	96.53	96.49
**Scenario C**								
[7,14)	99.35	98.52			99.24	0.00	98.76	96.10
[14,21)	99.19	98.22			96.71	22.96	98.34	93.92
[21,28)	99.26	98.66			98.83	85.93	98.30	97.53
[28,35)	99.67	98.79			99.40	98.11	98.51	98.56
[35,42)	99.57	98.50	23.85	87.97	99.51	98.36	98.23	98.34
[0,307]	97.60	95.89	95.36	94.39	97.95	92.67	96.00	96.30

Table C. Mean width of 95 percent Bayesian credible intervals of effective population size from all inferential techniques in each data scenario for 500 simulations in scenarios A, B, and C. Time periods span approximately from first simulated sampling time to 90th percentil of historic reporting delays, 41 days. The final time period for each simulation scenario spans from the first to last sampling time simulated.

	Retrospective		Real-Time Inference					
Time Period (days)	BNPR	BNPR PS	Trunc. BNPR	Tunc. BNPR PS	BNPR	BNPR PS	Delay-Aware BNPR PS (RP offset implementation)	BNPR PS (RP covariate implementation)
**Scenario A**								
[0,7)	39.06	23.68			138.99	16.69	83.81	74.24
[7,14)	29.61	19.29			69.37	21.83	38.36	44.73
[14,21)	27.18	17.09			36.48	23.90	25.08	25.85
[21,28)	24.75	15.41			29.31	22.69	19.60	20.60
[28,35)	22.63	14.10			25.77	20.27	16.60	17.88
[35,42)	20.67	12.95	31.91	23.54	23.06	17.94	14.50	15.69
[0,154]	17.74	10.73	16.35	10.00	20.21	12.57	12.37	13.00
**Scenario B**								
[7,14)	34.39	19.47			86.69	20.78	33.97	51.30
[14,21)	33.87	19.78			61.89	19.65	25.46	36.15
[21,28)	35.36	20.72			45.16	21.07	23.40	26.70
[28,35)	37.55	22.12			41.90	22.89	23.98	24.98
[35,42)	39.98	23.83	64.73	33.94	42.89	24.92	25.28	25.56
[0,228]	30.83	18.40	31.57	18.66	33.77	19.48	19.54	20.67
**Scenario C**								
[7,14)	71.12	45.30			174.69	42.27	79.11	114.94
[14,21)	69.30	46.56			107.61	46.78	60.30	73.87
[21,28)	67.87	45.53			79.86	51.78	53.39	55.21
[28,35)	64.83	43.03			71.50	50.95	48.45	49.27
[35,42)	61.57	39.92	73.03	53.62	65.93	47.69	43.52	44.63
[0,307]	47.19	28.39	45.18	27.16	50.51	31.19	30.12	31.94

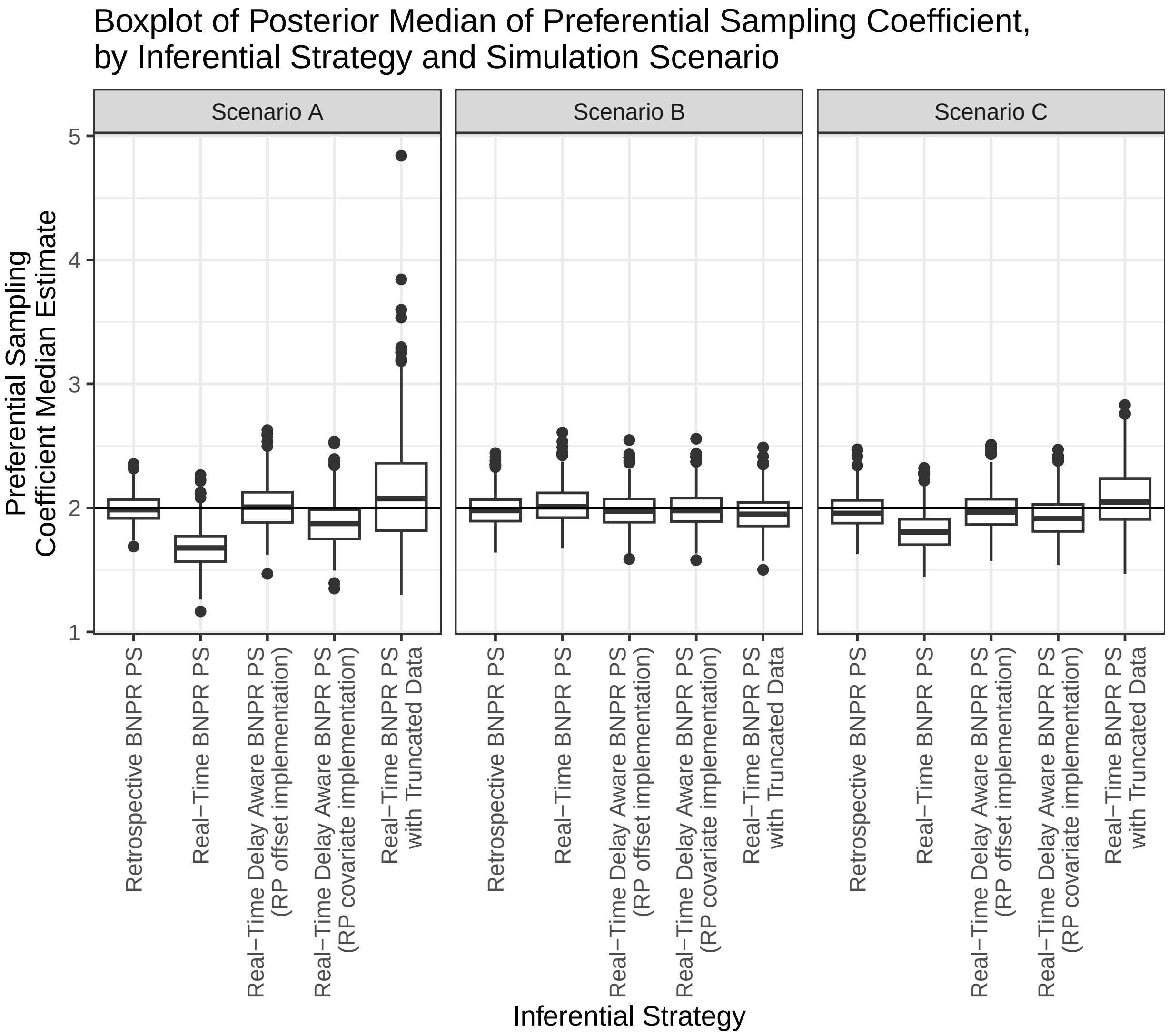

Fig F. Median preferential sampling coeffecient estimate from simulations by estimation strategy and simulation scenario.

1.2 Simulation with Santa Clara County reporting delays (more extreme delays than Washington state)

All simulation details are identical to the simulations in the main manuscript, with a difference in reporting probabilities. Here we use the empirical reporting delays of Santa Clara County as the reporting probabilities. The 90th percentile of reporting delays is 54 for Santa Clara County, while Washington state's was 41. Since these delays are more extreme this serves to help investigate how more extreme delays affect the analysis.

1.2.1 Results from last simulation in each scenario

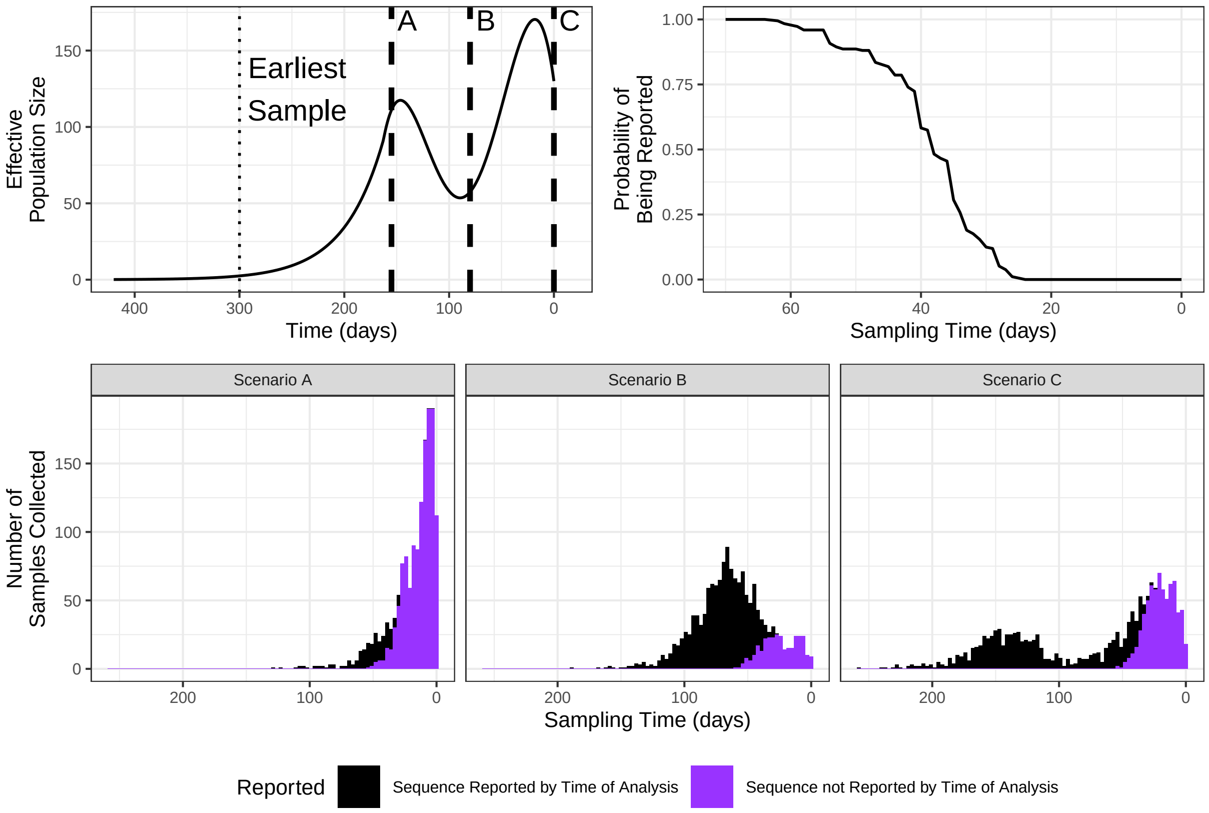

Fig G. Three panel plot providing simulation details: effective population trajectories (upper left plot), reporting probability by sampling time (upper right plot) obtained from Santa Clara County empirial cumulative distibution, and histograms of sampling times from the last simulation of in each simulation scenario colored by whether sample was reported by time of analysis (bottom plots). Each simulation scenario had a different time zero, i.e. time of latest sample (dashed lines). The earliest sampling time in each scenario was at the same point in the trajectory (dotted line).

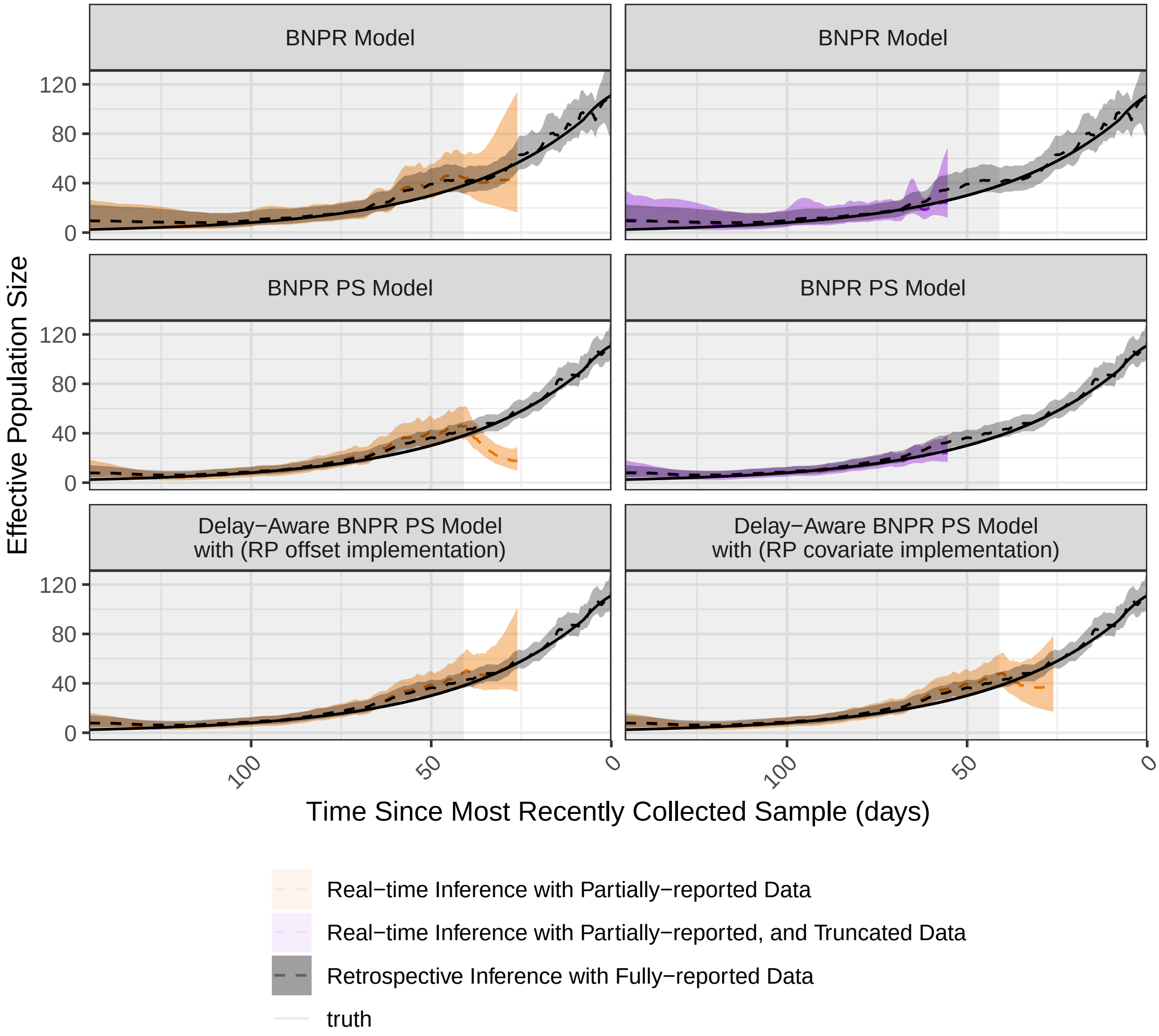

Fig H. Comparison of phylodynamic estimation methods of effective population size trajectory for three different simulated data scenarios from the scenario A trajectory.

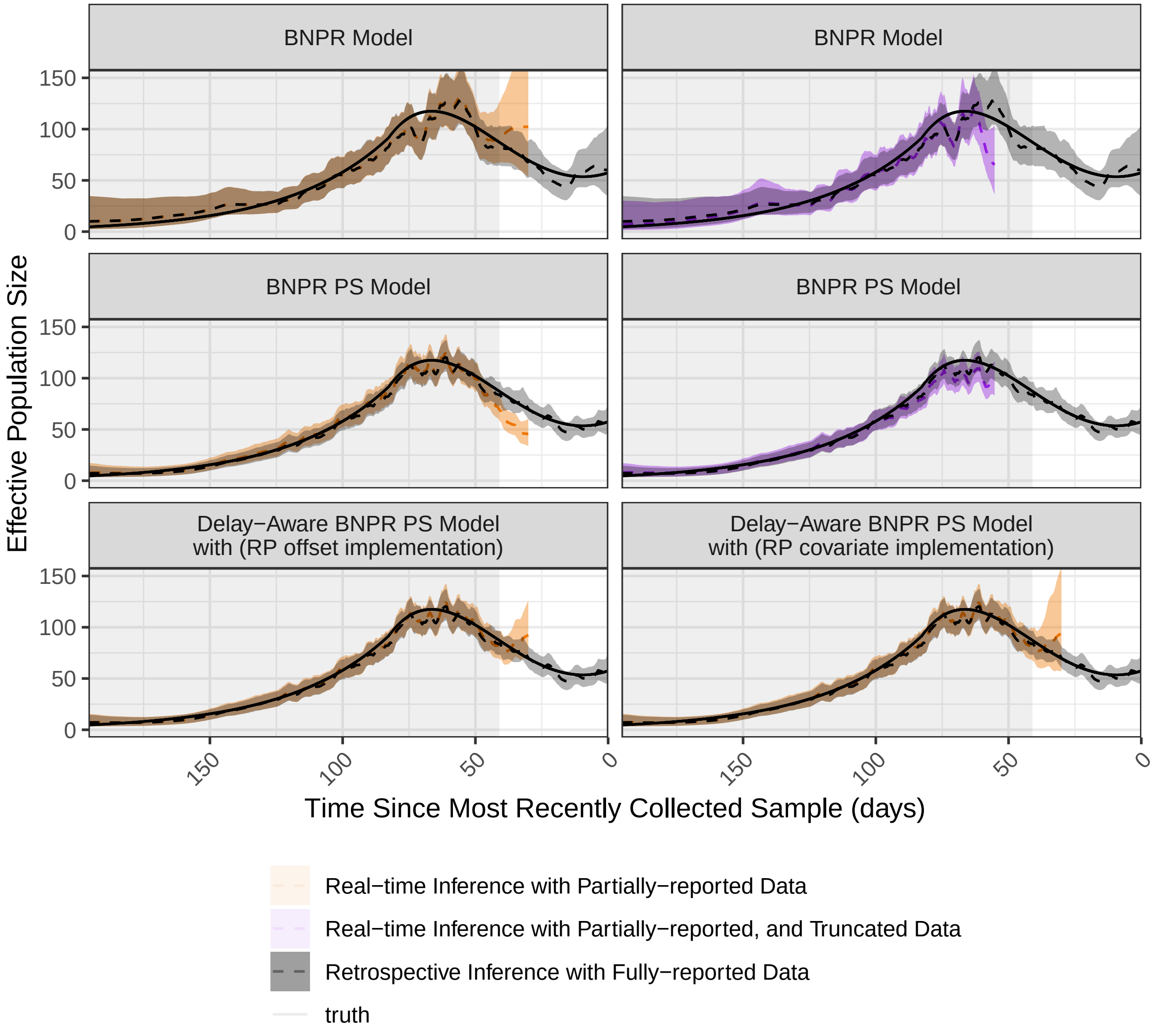

Fig I. Comparison of phylodynamic estimation methods of effective population size trajectory for three different simulated data scenarios from the scenario B trajectory.

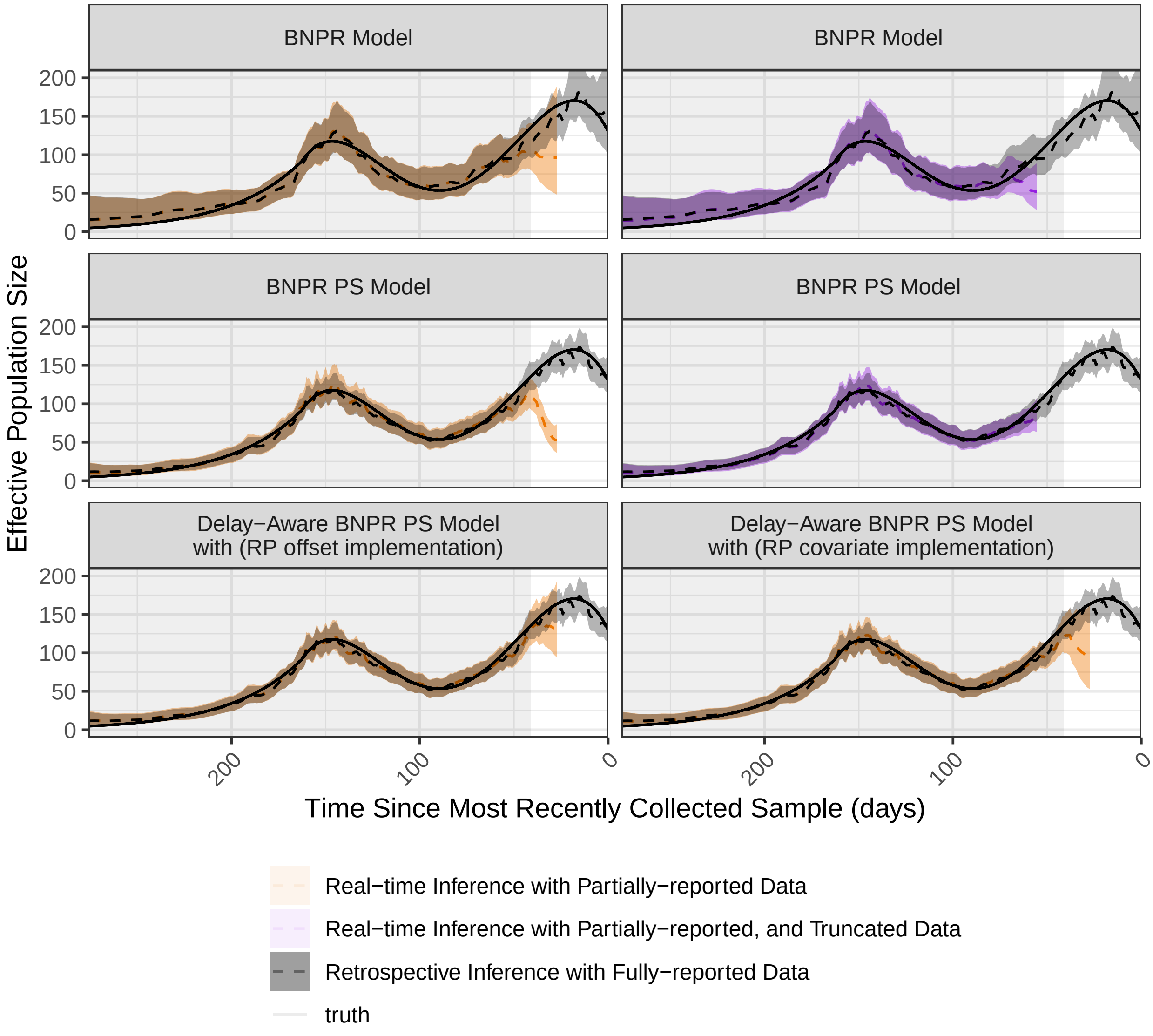

Fig J. Comparison of phylodynamic estimation methods of effective population size trajectory for three different simulated data scenarios from the scenario C trajectory.

1.2.2 Performance metrics across all simulations

Table D. Mean relative deviation of effective population size from all estimation techniques in each data scenario for 500 simulations in scenarios A, B, and C. Time periods span from first simulated sampling time to 90th percentile of historic reporting delays, 55 days. The final time period for each simulation scenario spans from the first to last sampling time simulated.

	Retrospective		Real-Time Inference					
Time Period (days)	BNPR	BNPR PS	Trunc. BNPR	Tunc. BNPR PS	BNPR	BNPR PS	Delay-Aware BNPR PS (RP offset imple-men-ta-tion)	BNPR PS (RP covari-ate imple-men-ta-tion)
**Scenario A**								
[0,7)	-0.01	0.00			-0.36	-0.96	0.07	-0.50
[7,14)	0.01	0.00			-0.46	-0.95	0.00	-0.53
[14,21)	0.00	0.00			-0.38	-0.90	-0.02	-0.50
[21,28)	0.00	0.00			-0.23	-0.68	-0.04	-0.35
[28,35)	0.00	-0.01			-0.14	-0.51	-0.02	-0.24
[35,42)	0.00	-0.01			-0.03	-0.16	0.00	-0.06
[42,49)	0.01	-0.01			0.01	0.04	0.01	0.02
[49,56)	0.01	-0.01	-0.35	-0.12	0.01	0.06	-0.01	0.02
[0,156]	0.12	0.05	0.11	0.02	0.10	-0.01	0.04	0.01
**Scenario B**								
[21,28)	0.01	0.00			0.39	-0.54	0.20	0.18
[28,35)	0.00	0.00			0.22	-0.48	0.06	0.07
[35,42)	0.00	0.00			0.08	-0.27	-0.01	0.00
[42,49)	0.00	0.00			0.01	-0.08	0.00	0.00
[49,56)	0.00	0.00	-0.36	-0.07	0.00	-0.03	0.00	0.00
[0,229]	0.15	0.06	0.13	0.08	0.17	0.06	0.07	0.07
**Scenario C**								
[14,21)	-0.01	0.01			-0.06	-0.84	0.18	-0.26
[21,28)	0.00	0.01			-0.18	-0.67	-0.03	-0.27
[28,35)	0.00	0.00			-0.16	-0.55	-0.04	-0.23
[35,42)	0.00	0.00			-0.08	-0.28	-0.01	-0.10
[42,49)	0.00	0.00			-0.03	-0.08	0.00	-0.02
[49,56)	0.00	0.00	-0.46	-0.11	0.00	-0.02	0.00	-0.01
[0,307]	0.14	0.07	0.12	0.05	0.13	0.04	0.07	0.05

Table E. Pecent of 95 percent Bayesian credible intervals that covered the true effective population size, from all estimation techniques in each data scenario for 500 simulations in scenarios A, B, and C. Time periods span from first simulated sampling time to 90th percentil of historic reporting delays, 55 days. The final time period for each simulation scenario spans from the first to last sampling time simulated.

	Retrospective		Real-Time Inference					
Time Period (days)	BNPR	BNPR PS	Trunc. BNPR	Tunc. BNPR PS	BNPR	BNPR PS	Delay-Aware BNPR PS (RP offset implementation)	BNPR PS (RP covariate implementation)
**Scenario A**								
[0,7)	99.13	97.97			100.00	0.00	100.00	100.00
[7,14)	99.20	98.48			100.00	0.00	100.00	100.00
[14,21)	99.25	98.75			100.00	0.00	100.00	90.91
[21,28)	99.19	98.74			99.20	0.00	99.70	90.16
[28,35)	99.45	99.12			97.80	3.30	98.05	88.91
[35,42)	99.54	98.88			98.56	74.88	98.26	95.61
[42,49)	99.46	98.55			99.57	98.32	98.11	98.51
[49,56)	99.13	98.56	81.81	93.91	99.23	98.15	98.47	98.69
[0,156]	98.36	96.71	97.42	96.68	98.83	89.72	97.57	97.10
**Scenario B**								
[21,28)	99.70	99.62			100.00	0.00	96.04	100.00
[28,35)	99.45	99.35			99.95	0.00	99.02	99.98
[35,42)	99.27	98.84			98.89	14.36	98.89	98.90
[42,49)	99.09	99.02			99.11	85.62	98.99	98.64
[49,56)	99.25	99.14	44.05	92.03	99.23	97.53	99.04	99.11
[0,229]	97.83	96.38	96.09	94.74	98.05	88.56	96.44	96.49
**Scenario C**								
[14,21)	98.72	98.54			100.00	0.00	100.00	100.00
[21,28)	99.80	99.21			99.53	0.00	99.37	95.91
[28,35)	99.31	98.59			98.27	0.00	98.33	89.99
[35,42)	99.50	98.87			97.12	20.17	98.43	91.60
[42,49)	99.68	99.50			99.19	90.77	98.82	98.35
[49,56)	99.19	98.97	32.32	92.99	99.42	98.84	99.08	99.16
[0,307]	97.86	96.15	95.46	96.48	98.32	91.67	96.77	96.61

Table F. Mean width of 95 percent Bayesian credible interval of effective population size from all estimation techniques in each data scenario for 500 simulations in scenarios A, B, and C. Time periods span from first simulated sampling time to 90th percentil of historic reporting delays, 55 days. The final time period for each simulation scenario spans from the first to last sampling time simulated.

	Retrospective		Real-Time Inference					
Time Period (days)	BNPR	BNPR PS	Trunc. BNPR	Tunc. BNPR PS	BNPR	BNPR PS	Delay-Aware BNPR PS (RP offset implementation)	BNPR PS (RP covariate implementation)
**Scenario A**								
[0,7)	38.90	23.77			530.38	9.86	583.96	208.03
[7,14)	29.59	19.36			262.33	8.94	306.41	120.18
[14,21)	27.00	17.13			164.57	10.44	155.74	77.54
[21,28)	24.62	15.46			99.80	16.86	63.91	54.50
[28,35)	22.49	14.08			66.95	17.33	39.84	41.11
[35,42)	20.59	12.94			34.46	20.61	24.48	24.51
[42,49)	18.84	11.93			24.46	20.31	17.88	18.87
[49,56)	17.22	10.96	30.12	24.04	21.32	17.39	14.60	15.76
[0,156]	17.60	10.71	15.91	9.87	20.76	12.11	13.27	13.47
**Scenario B**								
[21,28)	34.80	20.79			153.01	23.21	60.86	91.86
[28,35)	36.89	22.20			116.57	22.09	43.01	74.10
[35,42)	39.54	23.89			72.47	24.03	30.70	43.77
[42,49)	41.89	25.81			50.06	27.78	28.68	30.44
[49,56)	43.87	27.80	65.45	36.93	47.67	30.36	29.88	30.25
[0,229]	30.73	18.44	30.96	18.00	35.40	20.32	20.10	21.98
**Scenario C**								
[14,21)	68.83	46.38			375.50	28.30	273.68	200.07
[21,28)	67.69	45.33			225.11	38.12	128.97	147.24
[28,35)	64.90	42.95			169.17	36.12	88.34	114.28
[35,42)	61.57	39.90			101.09	41.18	58.65	66.72
[42,49)	57.18	36.53			68.31	43.16	45.50	45.75
[49,56)	53.04	33.49	63.02	48.23	59.05	39.49	38.77	39.00
[0,307]	47.22	28.35	44.08	27.42	50.66	30.03	30.76	32.15

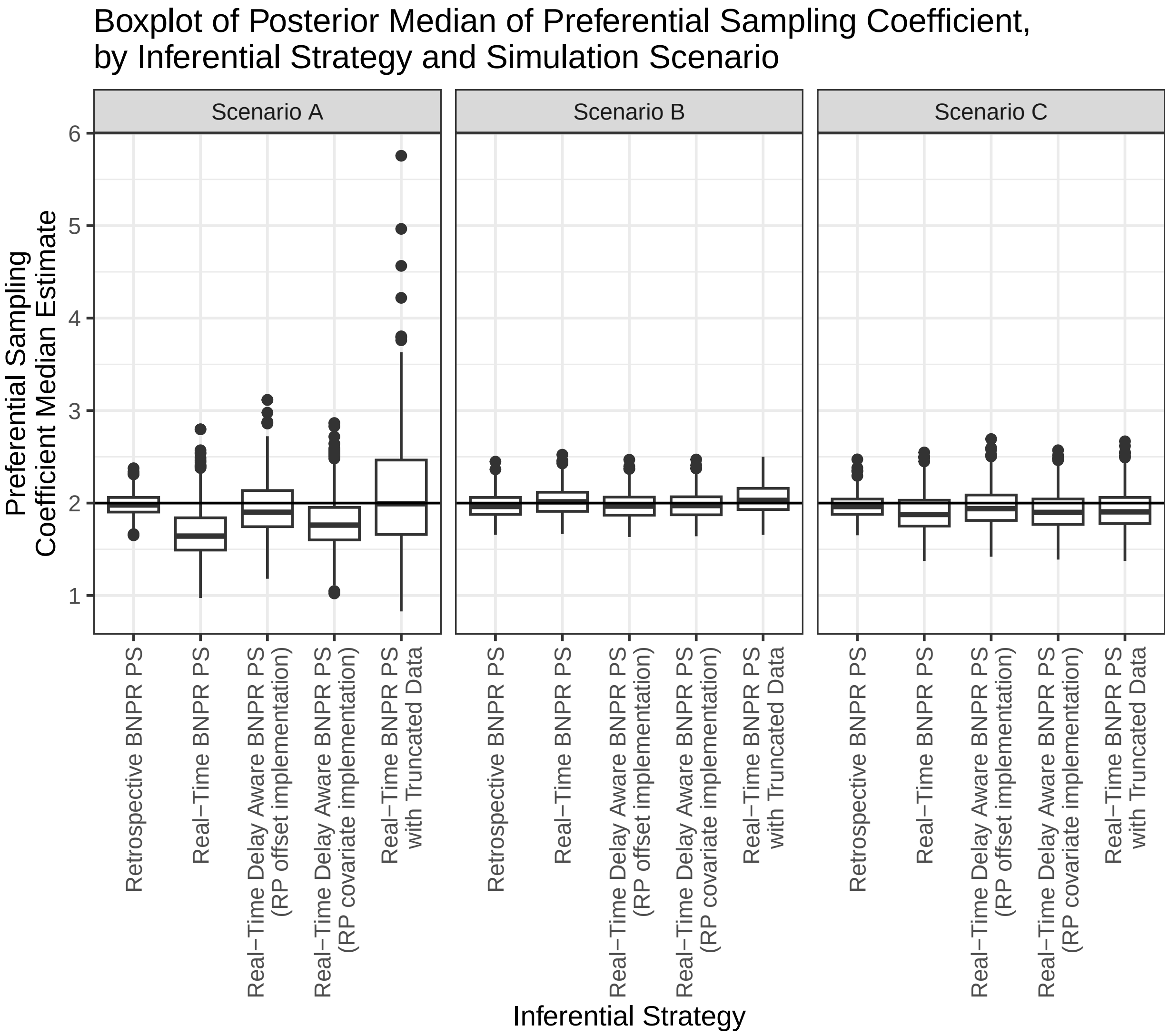

Fig K. Median preferential sampling coeffecient estimate from simulations by estimation strategy and simulation scenario.

2 Real data investigation: Washinton state COVID dynamics

2.1 Sequences from GISAID

Data Availability

GISAID Identifier: EPI_SET_240619zd
doi: 10.55876/gis8.240619zd

All genome sequences and associated metadata in this dataset are published in GISAID's EpiCoV database. To view the contributors of each individual sequence with details such as accession number, Virus name, Collection date, Originating Lab and Submitting Lab and the list of Authors, visit 10.55876/gis8.240619zd

Data Snapshot

- EPI_SET_240619zd is composed of 500 individual genome sequences.
- The collection dates range from 2021-02-01 to 2021-08-01;
- Data were collected in 1 countries and territories;
- All sequences in this dataset are compared relative to hCoV-19/Wuhan/WIV04/2019 (WIV04), the official reference sequence employed by GISAID (EPI_ISL_402124). Learn more at https://gisaid.org/WIV04.

Table G. BEAST modeling details for each real data scenario for Washington State samples reported to GISAID. All unnoted specifications were left as BEAST defaults.

	Full Scenario	Observed Scenario	Truncated Scenario
Data	Sampled before 2021-08-01, inclusive	Reported before 2021-08-01, inclusive	Sampled before 2021-06-21, inclusive
Models			
Substitution	HKY	HKY	HKY
Clock type	strict	strict	strict
Coalescent	Bayesian Skygrid	Bayesian Skygrid	Bayesian Skygrid
# of parameters	50	50	50
Last transition	1.63 years	1.52 years	1.6 years
Priors			
Kappa	LogNormal(1, 1.25)	LogNormal(1, 1.25)	LogNormal(1, 1.25)
Frequencies	Dirichlet(1, 1)	Dirichlet(1, 1)	Dirichlet(1, 1)
Clock rate	Unif(3e-4, 1.1e-3)	Unif(3e-4, 1.1e-3)	Unif(3e-4, 1.1e-3)
Root height	None (tree prior only)	None (tree prior only)	None (tree prior only)
Skygrid precision	Gamma(0.001, 1000)	Gamma(0.001, 1000)	Gamma(0.001, 1000)
MCMC options			
Chain length	2e+07	2e+07	2e+07
Burn in	2500000	2500000	2500000
Log every	2000	2000	2000
Seed	-	-	-

2.2 BEAST modeling details for real data investigation: Washington State

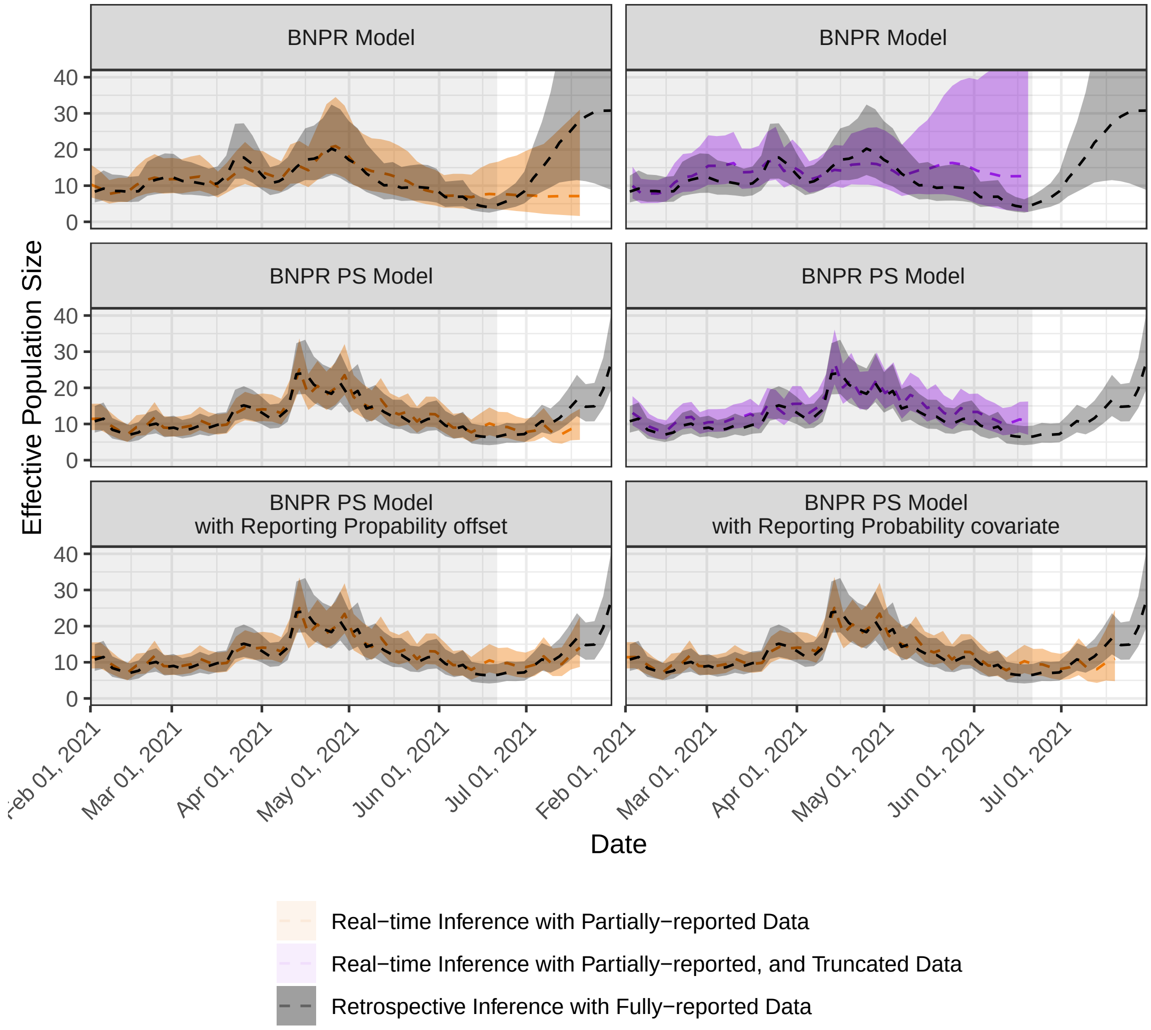

Fig L. Comparison of phylodynamic estimation methods of effective population size trajectory for Washington State SARS-CoV-2 sequences.

3 Investigation of reporting probability options

3.1 Options for calculating reporting probabilities

We discuss four options to calculate reporting probabilities from "historic reporting delays"

1. Use empirical cdf of delays from recently **observed reported** sequences
2. Use empirical cdf of delays from recently **observed sampled** sequences (censored)
3. Use Kaplan Meier estimates from recently **observed delays** and **all recent sampling times** (requires all sampling times are known, even censored ones). Note this is not appropriate for our case because we do not know about sampled sequences that were not observed.
4. Use empirical cdf of delays from **all recently sampled** sequences (retrospective option only - considered for comparison)

Another consideration is what is meant by "recently". The idea is to use recent data to estimate the current reporting probabilities. This requires investigation of reporting delay behavior and how it is changing overtime. The window of time used to define "recent" data should extend back from current time, "time zero" to a time where the reporting delay behavior appears to be relatively similar/consistent.

Here we will compare the results from defining "recently" to be one month versus three months.

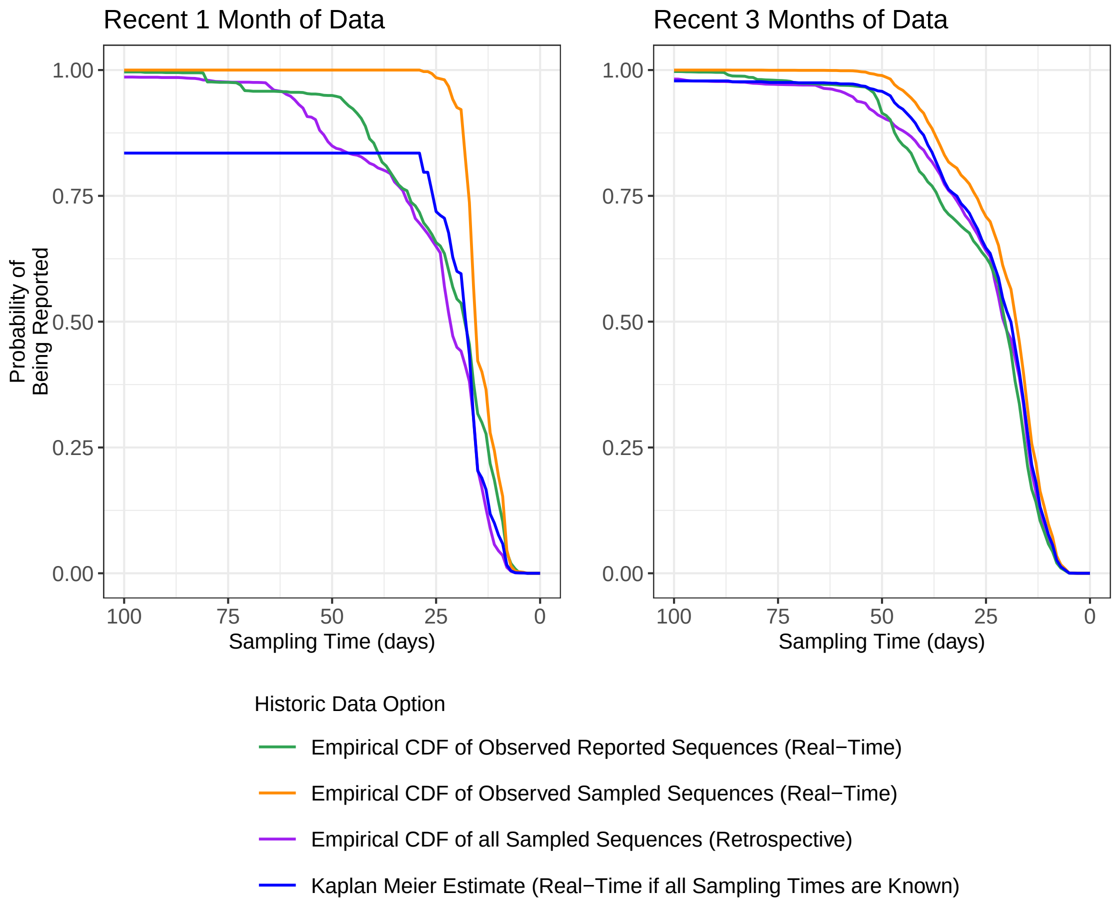

Fig M. Reporting probabilities obtained from different methods.

3.2 Comparison of inference with simulated data with different historic data options

It is important to understand how our proposed delay-aware BNPR PS model performs when the reporting probabilities are misspecified. Everything in the following simulations is the same as in the main text, except for the true reporting probabilities and the reporting delays used in the inference. For the true reporting probabilities we chose to use the empirical cdf of the reporting delays for the past one month of all sampled sequences (regardless of if they were observed).

For the inference with our proposed delay-aware BNPR PS model we chose to consider two realistic choices of misspecified reporting probabilities:

- Empirical cdf of delays from most recent one month of observed reported sequences (minor misspecification). The recently reported sequences are the ones we propose to be used since they will not suffer from the censoring that recently sampled sequences will.
- Empirical cdf of delays from most recent one month of observed samples sequences (major misspecifiaction due to censoring). This may be a tempting choice to use because recently sampled sequences would be more representative of recent reporting delay behavior, but this data would suffer from censoring due to reporting delays.

For both cases we chose to use the most recent month of data. We chose this because a smaller time window for the "historic delays" means fewer samples. This decision is situation specific, and for our case we believe this provides more misspecification of reporting probabilities than a larger window such as three months.

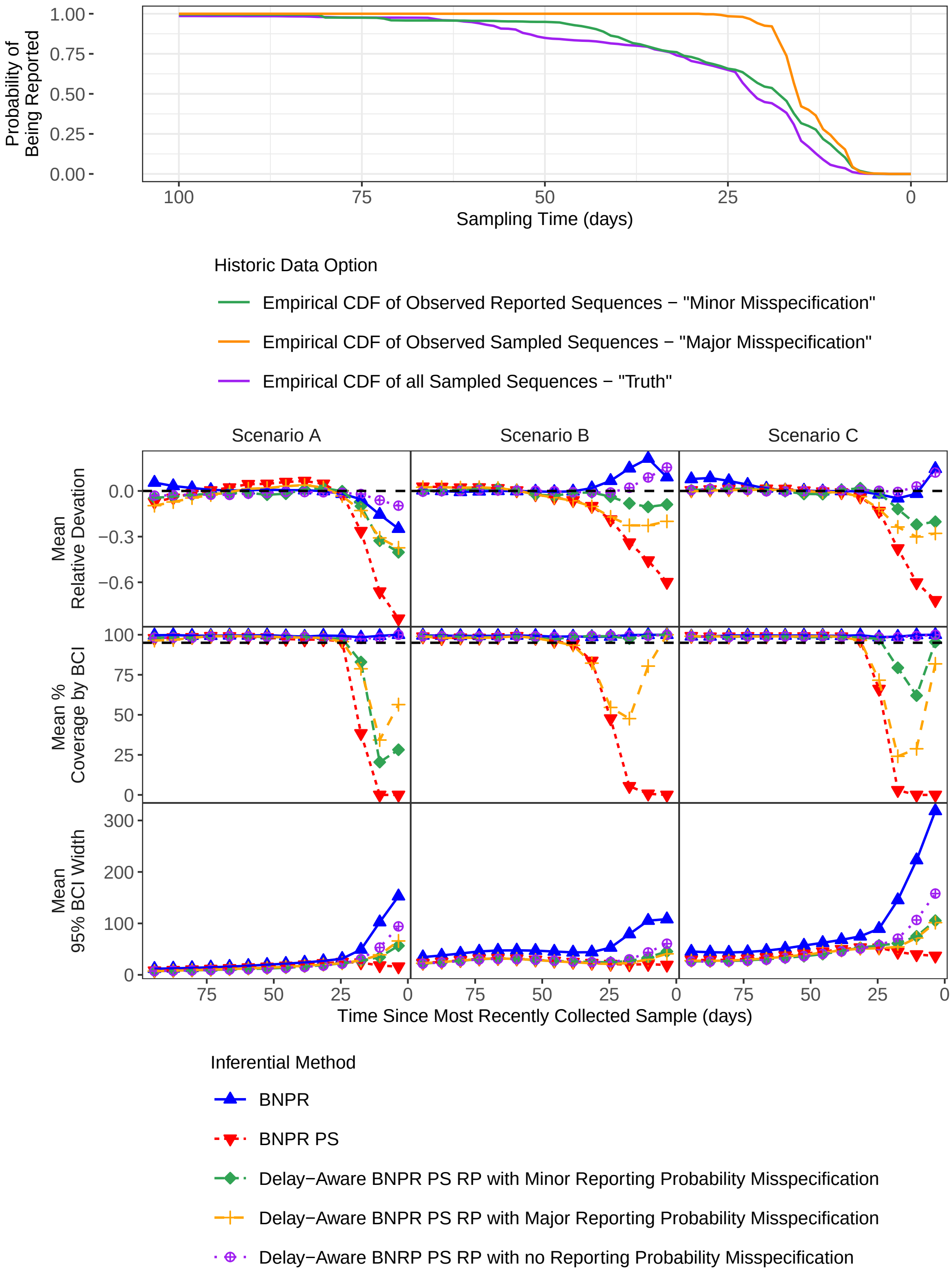

Fig N. The top plot shows three different reporting probability specifications. The bottom plot has the inference results from the methods discussed in the main manuscript, plus inference results from the delay-aware BNPR PS model misspecifications of reporting probabilities.

Figure N shows the reporting probabilities used as the “truth” in the simulation (shown in purple), “minor misspecification” of using recently reported sequences (shown in green), and the “major misspecification” of using recently observed sampled sequences (shown in orange). In the second plot of this figure we also see the same performance evaluation metrics previously discussed.

The purple setting is when the true reporting probabilities are used in with our proposed delay-aware BNPR PS model, so these results are the same as what we previously saw in the simulations from the main manuscript. In the new settings with the major and minor reporting probabilities misspecifications we see our model still perform better than the BNPR PS model – having lower bias and higher credible interval coverage. We also see both of these misspecified models have competitive bias relative to the BNPR model, excpet in the low observed data setting of Scenario A. When comparing the models with major and minor misspecifications we do see that the more misspecification of reporting probabilities results in more bias and lower coverage.

These simulations support that our proposed delay-aware BNPR PS model, where reporting probabilities are obtained through the empirical cdf of recent delays, should be used when one believes that their sample of “recent reporting delays” is similar to the current reporting delay behavior. Another finding is that the delays of recently reported sequences should be used instead of recently sampled sequences because the censoring results in consistently worse inference. Finally, these results also indicate that in low data situations with misspecification of the reporting probabilities, the BNPR model would be the better choice because its low precision results in better coverage.

3.3 Inference with Washington data with different historic data options

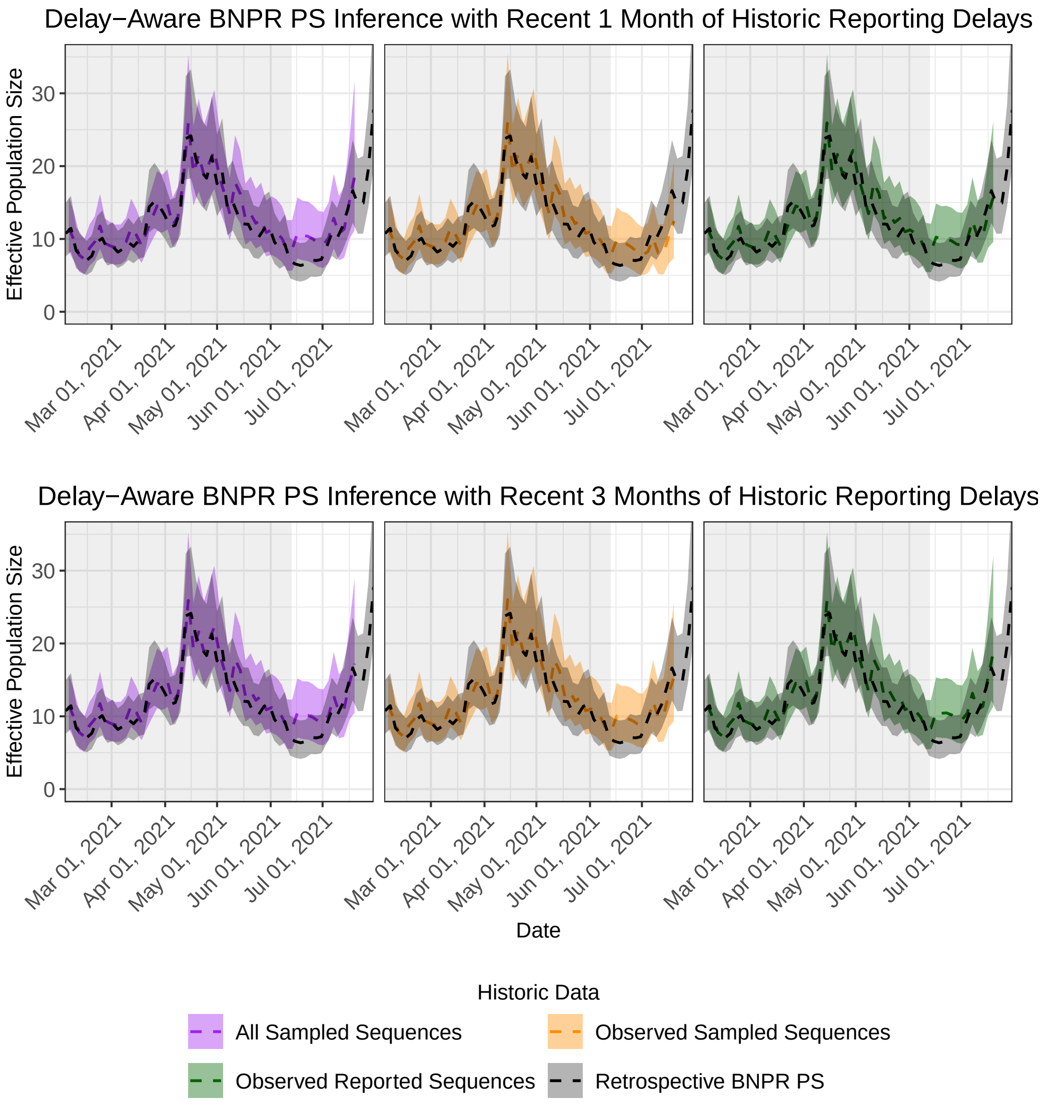

Fig O. Washinton inference with reporting probabilities obtained from different methods.

The results shown in Figure O are consistent with those seen in our simulations. Using the censored reporting delays of recently sampled sequences are used the inference is least similar to that of the retrospective BNPR PS model with no reporting delays in data. We also see with this scenario that inference with the recently observed reported (green) and sampled (orange) sequences is least similar to the retrospective BNPR PS mode when using only the most recent month of data, relative to the last three months of data. Fortunately, there is little difference

when using the delays from the previous three months versus one month of reported sequences, showing our results are not too sensitive to minor differences in the reporting probabilities, visualized in Figure M.

4 Investigation of different phylogenies estimated using Washington data

In our investigations we assumed that the phylogenetic tree was known and used the maximum clade credibility tree obtained using TreeAnnotator from BEAST MCMC output. It is important to know how much the unaccounted phylogentic uncertainty from the tree affects our inference. Below are results where each figure is from a different tree.

The first tree is the maximum clade credibility tree obtained from the observed sequences, which was used in the real data investigation in the main text, it is repeated here to make comparisons easier. The other five plots are from five randomly selected trees from the posterior provided by BEAST on only the observed sequences. The retrospective analysis with the BNPR PS model shown in gray in each of the following plots was done with the maximum clade credibility tree from all 500 sequences (observed and unobserved combined).

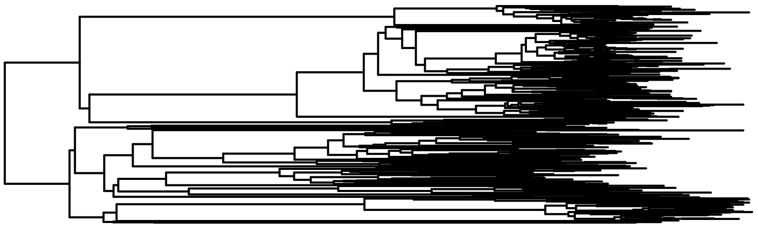

Fig P. Washinton Maximum clade credibility tree.

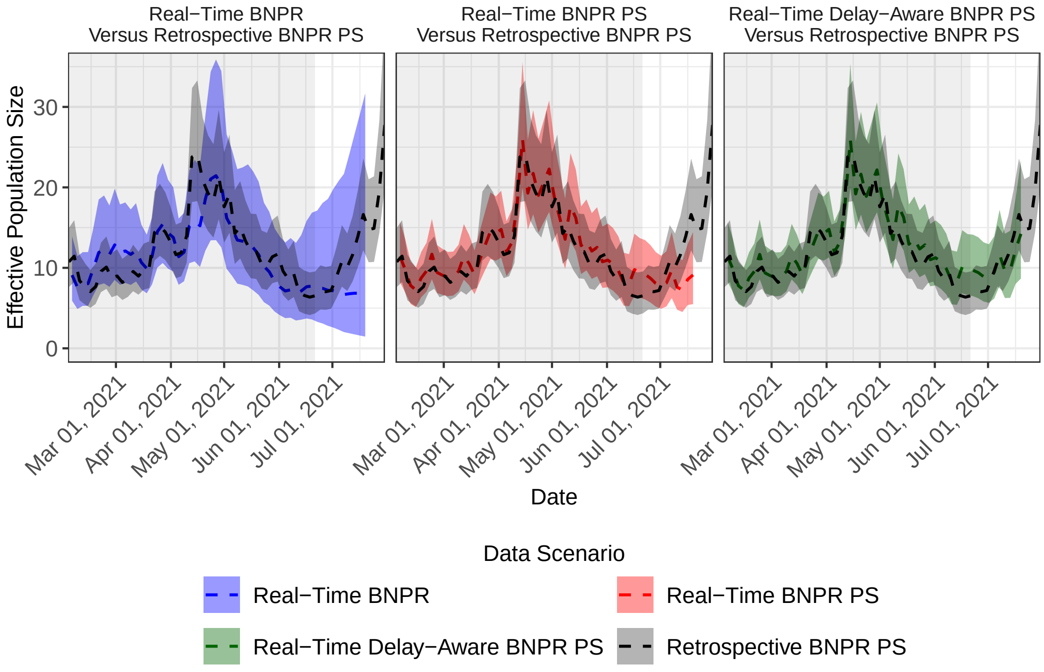

Fig Q. Washinton inference with maximum clade credibility tree.

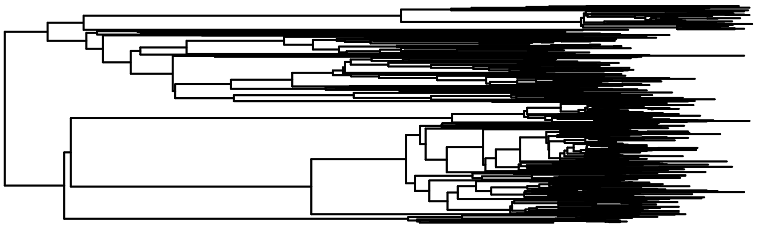

Fig R. Washinton randomly selected posterior tree 1.

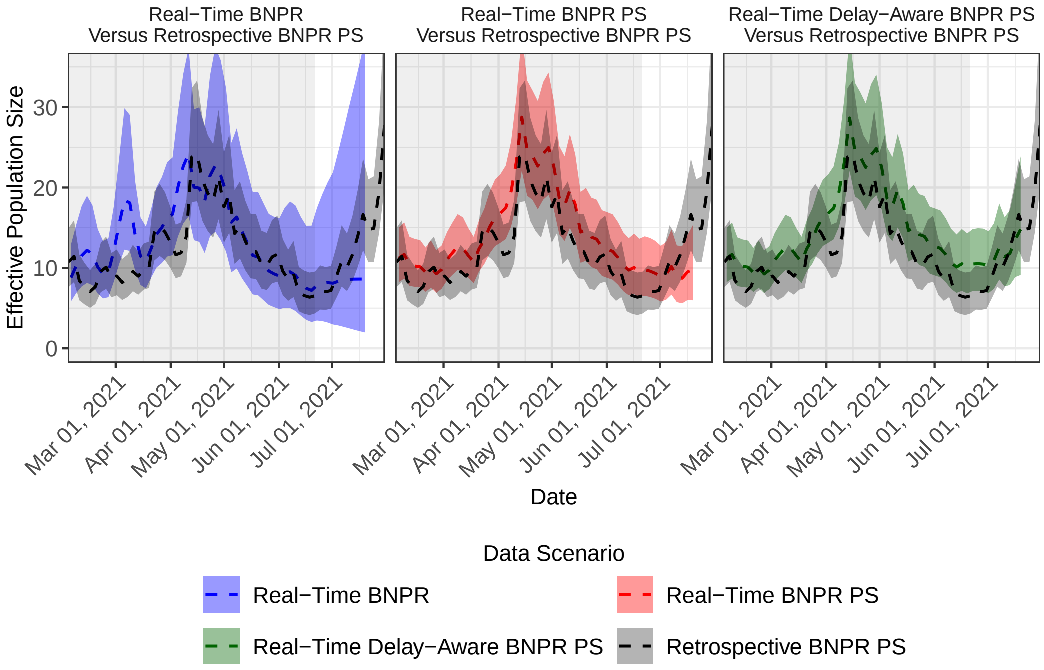

Fig S. Washinton inference with randomly selected posterior tree 1.

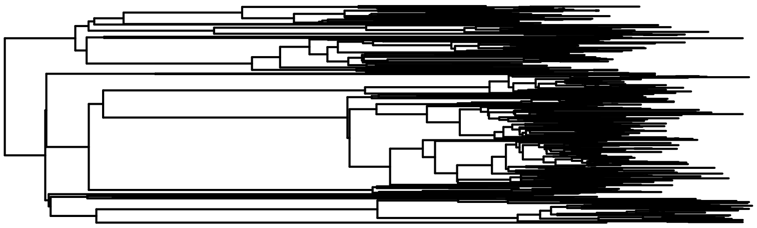

Fig T. Washinton randomly selected posterior tree 2.

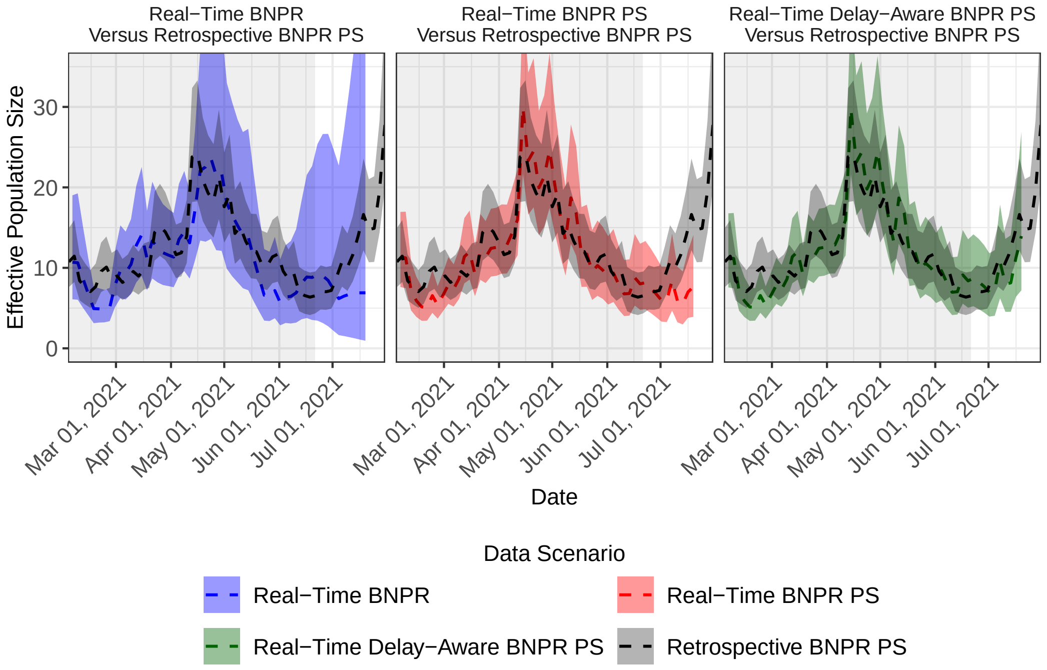

Fig U. Washinton inference with randomly selected posterior tree 2.

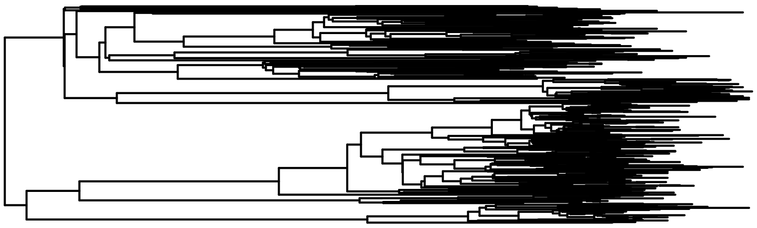

Fig V. Washinton randomly selected posterior tree 3.

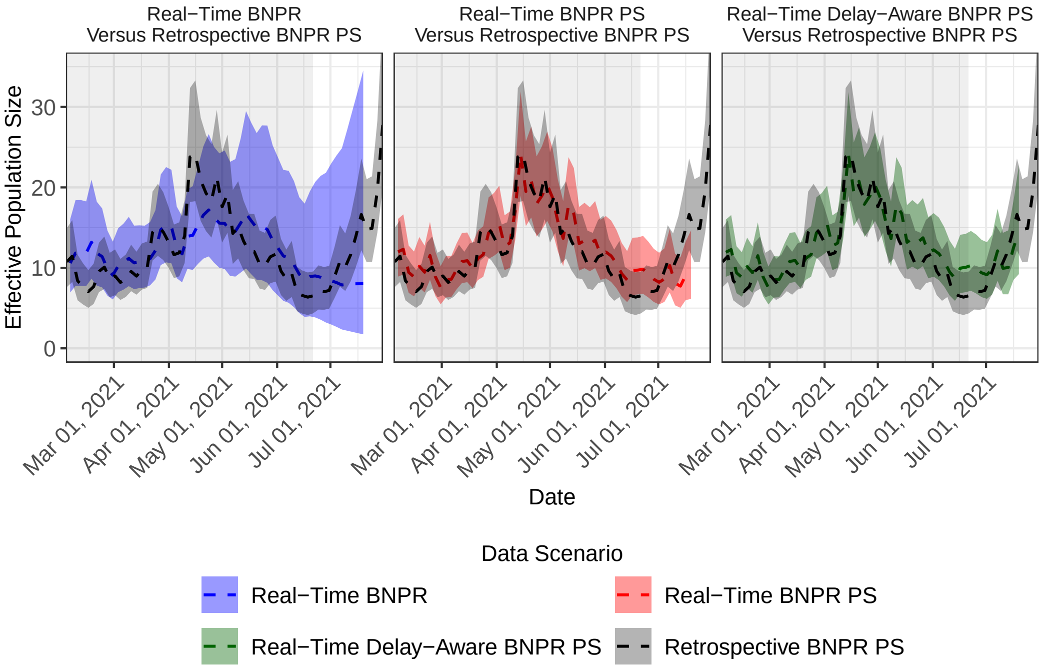

Fig W. Washinton inference with randomly selected posterior tree 3.

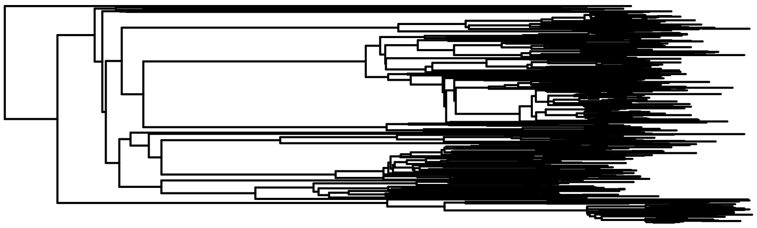

Fig X. Washinton randomly selected posterior tree 4.

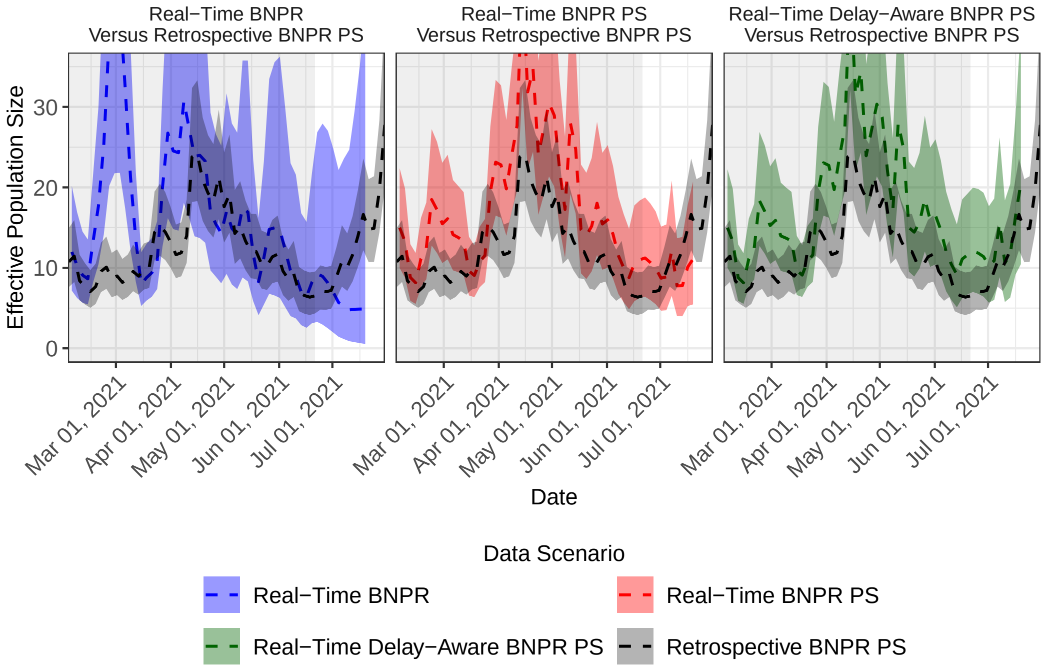

Fig Y. Washinton inference with randomly selected posterior tree 4.

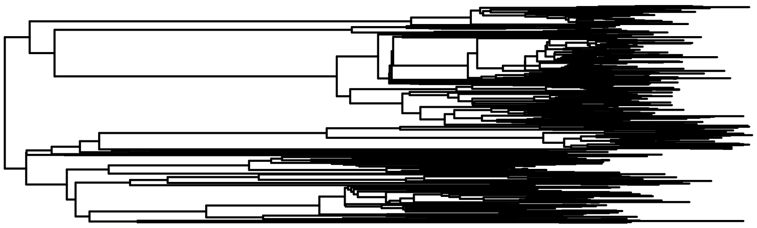

Fig Z. Washinton randomly selected posterior tree 5.

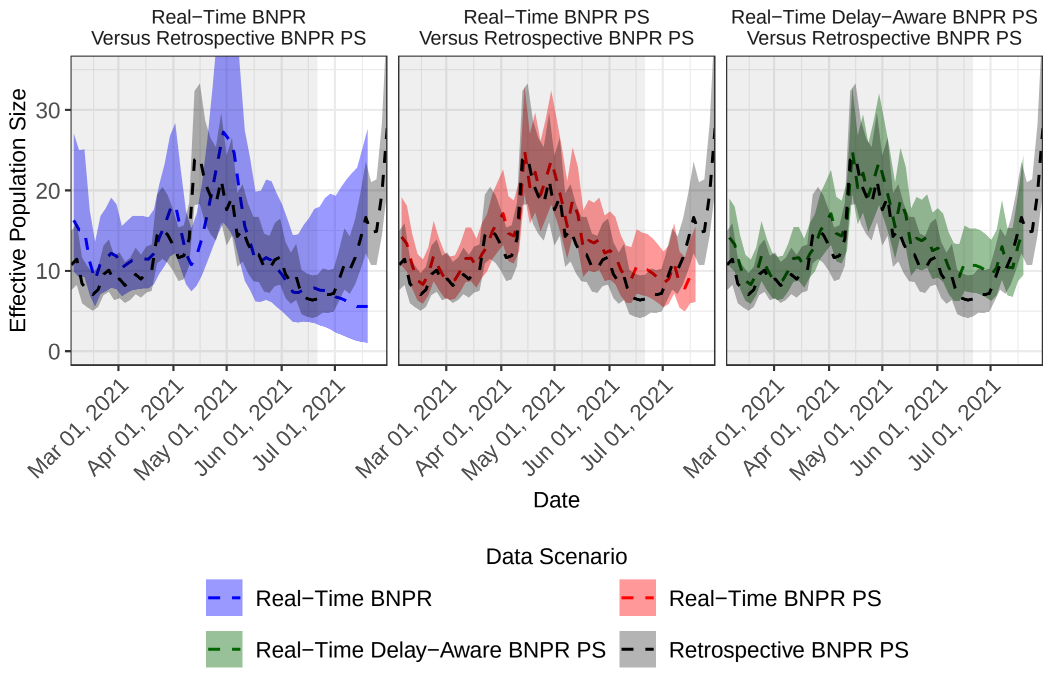

Fig AA. Washinton inference with randomly selected posterior tree 5.

The results from using the maximum clade credibility tree, used in the main analysis, and the

other five randomly selected posterior trees are all relatively similar, with the exception of tree 4. This suggests that we are underestimating effective population size uncertainty, which is expected. It is difficult to quantify the magnitude of this underestimation from just 5 posterior samples, but robustness of our inference to phylogenetic tree perturbation looks promising.
